# Validation of the Danish Psychosocial Questionnaire (DPQ) in a Swedish healthcare context

**DOI:** 10.1186/s12913-026-15083-z

**Published:** 2026-07-13

**Authors:** Alexander Agrell, Susanne Tafvelin, Thomas Clausen, Johan Simonsen Abildgaard, Jens Wahlström, Robert Lundmark

**Affiliations:** 1 Department of Psychology, Umeå University, Umeå, 901 87 Sweden; 2 National Research Center for the Work environment, Copenhagen, Denmark; 3 Copenhagen Business School, Copenhagen, Denmark; 4 Department of Epidemiology and Global Health, Umeå University, Umeå, Sweden; 5 Department of Health, Education and Technology, Luleå University of Technology, Luleå, Sweden

**Keywords:** Psychosocial work environment, Healthcare, Validation, Psychometric properties, Single-item, Confirmatory factor analysis

## Abstract

**Background:**

Healthcare organizations in Europe, including Sweden, face ongoing challenges in ensuring a good work environment for their personnel, which is essential for high-quality care and job satisfaction. The Danish Psychosocial Work Environment Questionnaire (DPQ) has proven effective in various contexts but has not yet been validated in Swedish healthcare settings. This study aims to validate a Swedish translation of the DPQ in a healthcare context. We also aim to assess its validity against a broader range of outcomes than previously explored and to evaluate the feasibility of a single-item version for practical use.

**Methods:**

The study was conducted within a public healthcare organization in northern Sweden and included 1,299 employees from four departments. The DPQ was translated into Swedish and subsequently administered, yielding a response rate of 48% at Time 1 (T1). Participants were randomly assigned to two groups: one group received the full DPQ at both time points, while the other received the full DPQ at T1 and a single-item version at Time 2 (T2). Reliability and validity were assessed using confirmatory factor analysis (CFA) whilst the evaluation of the single-item version of DPQ was done by a comparison of correlation coefficients.

**Results:**

The Swedish DPQ demonstrated good reliability and validity, with omega values > 0.70 for all subscales. CFA confirmed the multifactor structure of the instrument. High intercorrelations between some subscales indicated potential overlap. The single-item version showed significant differences in correlation coefficients compared to the full version, suggesting it may not be a suitable alternative.

**Conclusions:**

The Swedish translation of the DPQ is a reliable and valid tool for assessing the psychosocial work environment in healthcare. However, the single-item version may not provide an accurate representation of complex constructs. The study highlights the importance of comprehensive measures for improving working conditions and suggests further research in broader contexts.

**Supplementary Information:**

The online version contains supplementary material available at 10.1186/s12913-026-15083-z.

## Introduction

In Europe, healthcare organizations face ongoing challenges in providing a healthy and supportive work environment for their personnel [1], with Sweden being no exception [2]. A good work environment in healthcare is related to high quality of care [3], as well as higher job satisfaction and health among healthcare workers [4, 5]. Healthcare work can be stressful, with decisions that affect the well-being of others and exposure to others’ suffering from disease. At the same time, most healthcare organizations face challenges in terms of limited financial resources, staff shortages, and a high degree of turnover [6], a situation that has worsened in the aftermath of the COVID-19 pandemic [7]. Consequently, health indicators such as depression and burnout levels, as well as intentions to quit their jobs, are significantly higher among healthcare workers compared to other job groups [8].

Given the limited resources available, it has been argued that it is of vital importance to find efficient measures to improve conditions, and for this to be realized, an important first step is to identify where improvements are needed [9]. In other words, healthcare organizations that are provided with effective work environment assessment instruments can be more precise in targeting efforts to improve working conditions at specific problem areas. The data gathered from these instruments can also help healthcare organizations and decision-makers prioritize and allocate resources where they will be most beneficial. Furthermore, healthcare workers can use the instruments to plan and monitor the effects of interventions aimed at improving their work environment. However, at present, there are few up-to-date instruments for screening working conditions that have been adapted to and validated in a healthcare context. Additionally, it is essential that such instruments are rigorously tested in the specific context where they are intended to be used and evaluated from both a scientific and practical perspective [10]. Lastly, Swedish legislation requires organizations to regularly monitor and follow up on the work environment, including the psychosocial aspects [11, 12], making the need for instruments assessing these aspects essential.

The Danish Psychosocial Work Environment Questionnaire (DPQ) has proven to be a robust instrument for mapping working conditions in a variety of work contexts, including healthcare professionals among other professions [13]. It is a comprehensive, multi-theory-based questionnaire developed by the National Research Center for the Work environment in Denmark. Rather than being grounded in a single theoretical framework, the DPQ was developed to operationalize a broad range of established concepts within the psychosocial work environment and worker well-being [13]. This multifaceted approach was chosen to ensure flexibility and relevance across different contexts, addressing both the need for validated measures in research and evidence-based tools for workplace risk assessments. The DPQ is available in different versions: a full scientific version and a shortened workplace version for practical use in workplaces [14]. The constructs, also referred to as subscales, measured in DPQ are divided into five dimensions where one measures demands, two measure resources, one measures outcomes and one measures negative work acts (such as harassment and bullying). DPQ has continuously been shown to be a useful tool to assess and predict important outcomes such as depression and long-term sickness absence among workers [15].

Based on the above, the aim of the current study is to validate a Swedish translation of the workplace version of the DPQ in a Swedish healthcare context. Furthermore, we move beyond the original scale validation study [13] by including additional outcomes relevant to the healthcare context, including patient safety, turnover intention, health, and performance. We thereby adapt the questionnaire validation to fit the context by testing its applicability in relation to these healthcare-relevant outcomes. Additionally, including these healthcare-specific outcomes also makes it possible to examine and compare the prevalence of these indicators across units and institutions.

Since time is often critical for healthcare personnel, an initial screening tool should be quick to administer while still producing valid and reliable results, especially if the alternative is no screening at all. Therefore, a second aim of this study is to explore whether a single-item version of the subscales in the DPQ could be a viable option for both practice and research. Currently, although there have been calls for simple tools to measure the work environment for healthcare workers [16], to our knowledge, no such short screening instrument has been tested for reliability and validity among healthcare workers and previous research concerning single-item questions and the work environment is mainly focused on the single construct job satisfaction [17, 18]. Therefore, this study also provides a starting point for future examinations of the potential use of short-form questionnaires with single items for use in the healthcare context.

## Method

### Study design, setting and period

The study was conducted in a public healthcare organization in northern Sweden. The organization employs over 10,000 people, the vast majority of whom are employed in a healthcare profession [19]. Four different departments, a surgery center (highly specialized and time-critical precision work), a laboratory center (characterized by being highly technical and mainly non-patient-facing), a habilitation center (long-term patient relationships and multidisciplinary teamwork), and a primary healthcare center (providing broad frontline medical services), were invited to participate in the present validation study. These departments were selected because they provided a good representation of different geographical locations, professions, specialized and general care within the organization. The questionnaire was sent out to all 1,299 employees of these departments, which was estimated to be a sufficient sample size to ensure adequate statistical power for the analyses, assuming a response rate of approximately 50%. The initial data collection started at the end of November 2021 and was open for four weeks. During that time, five reminders were sent out to those who had not completed the survey. 624 employees responded to the questionnaire at Time 1 (T1), resulting in a response rate of 48%. See Table 1 for characteristics of the respondents including information about sex, mean age, level of education, profession and years spent working in the organization.

**Table 1:** Characteristics of respondents at T1

Characteristics *N*(%)	Surgery center	Laboratory	Habilitation center	Primary care	Total
Sex					
Female	165 (80,5%)	157 (78,1%)	93 (90,3%)	98 (85,2%)	513 (82,2%)
Male	38 (18,5%)	42 (20,9%)	9 (8,7%)	17 (14,8%)	106 (17,0%)
Other/missing	2 (1,0%)	2 (1.0%)	1 (1,0%)	0 (0,0%)	5 (0,8%)
Age M (sd)	41,5 (12,5)	46,3 (12,0)	49,1 (9,5)	47,4 (11,2)	45,4 (11,9)
Degree					
Primary School	1 (0,5%)	1 (0,5%)	1 (1,0%)	1 (0,9%)	4 (0,6%)
High school	49 (23,9%)	20 (10,0%)	4 (3,9%)	27 (23,5%)	100 (16,0%)
University	144 (70,2%)	147 (73,1%)	96 (93,2%)	86 (74,8%)	473 (75,8%)
PhD	9 (4,4%)	33 (16,4%)	1 (1,0%)	1 (0,9%)	44 (7,0%)
Missing	2 (1,0%)	0 (0,0%)	1 (1,0%)	0 (0,0%)	3 (0,5%)
Occupation					
Nurse	81 (39,5%)	12 (6,0%)	1 (1,0%)	34 (29,6%)	128 (20,5%)
Assistant nurse	44 (21,5%)	4 (2,0%)	-	22 (19,1%)	70 (11,2%)
Physician	34 (16,6%)	26 (12,9%)	-	10 (8,7%)	70 (11,2%)
Psychologist/Counselor	1 (0,5%)	-	38 (36,9%)	7 (6,1%)	46 (7,4%)
Manager	11 (5,4%)	18 (9,0%)	6 (5,8%)	11 (9,6%)	46 (7,4%)
Medical secretary	17 (8,3%)	7 (3,5%)	-	7 (6,1%)	31 (5,0%)
Care administrator	7 (3,4%)	7 (3,5%)	8 (7,8%)	11 (9,6%)	33 (5,3%)
Physiotherapist	2 (1,0%)	-	9 (8,7%)	9 (7,8%)	20 (3,2%)
Biomedical analyst/Lab technician	-	127 (63,2%)	-	-	127 (20,4%)
Occupational therapist	1 (0,5%)	-	18 (17,5%)	3 (2,6%)	22 (3,5%)
Other	7 (3,4%)	-	23 (22,3%)	1 (0,9%)	31 (5,0%)
Years in organization					
< 3 years	43 (21,0%)	29 (14,4%)	18 (17,5%)	20 (17,4%)	110 (17,6%)
3–13 years	92 (44,9%)	85 (42,3%)	40 (38,8%)	46 (40,0%)	263 (42,1%)
> 13 years	70 (34,1%)	86 (42,8%)	45 (43,7%)	48 (41,7%)	249 (39,9%)
Missing	-	1 (0,5%)	-	1 (0,9%)	2 (0,3%)

### Single-item version

The single-item version of the DPQ was developed through a stepwise reduction procedure. First, subscales with high intercorrelations were identified and, where substantial overlap was observed, subscales were removed to reduce redundancy between constructs while maintaining conceptual coverage of the instrument [20].

Subsequently, one representative item was selected from each of the remaining subscales based on statistical performance and theoretical relevance, resulting in a brief instrument intended to capture the core aspects of each construct while minimizing respondent burden.

The purpose of this single-item version was to explore its potential usability as a screening instrument in contexts where repeated measurements or limited response time make full-length questionnaires impractical. A more detailed description of the reduction procedure and item selection is provided in the Results section (Phase 4 – Instrument reduction).

**Fig. 1:**
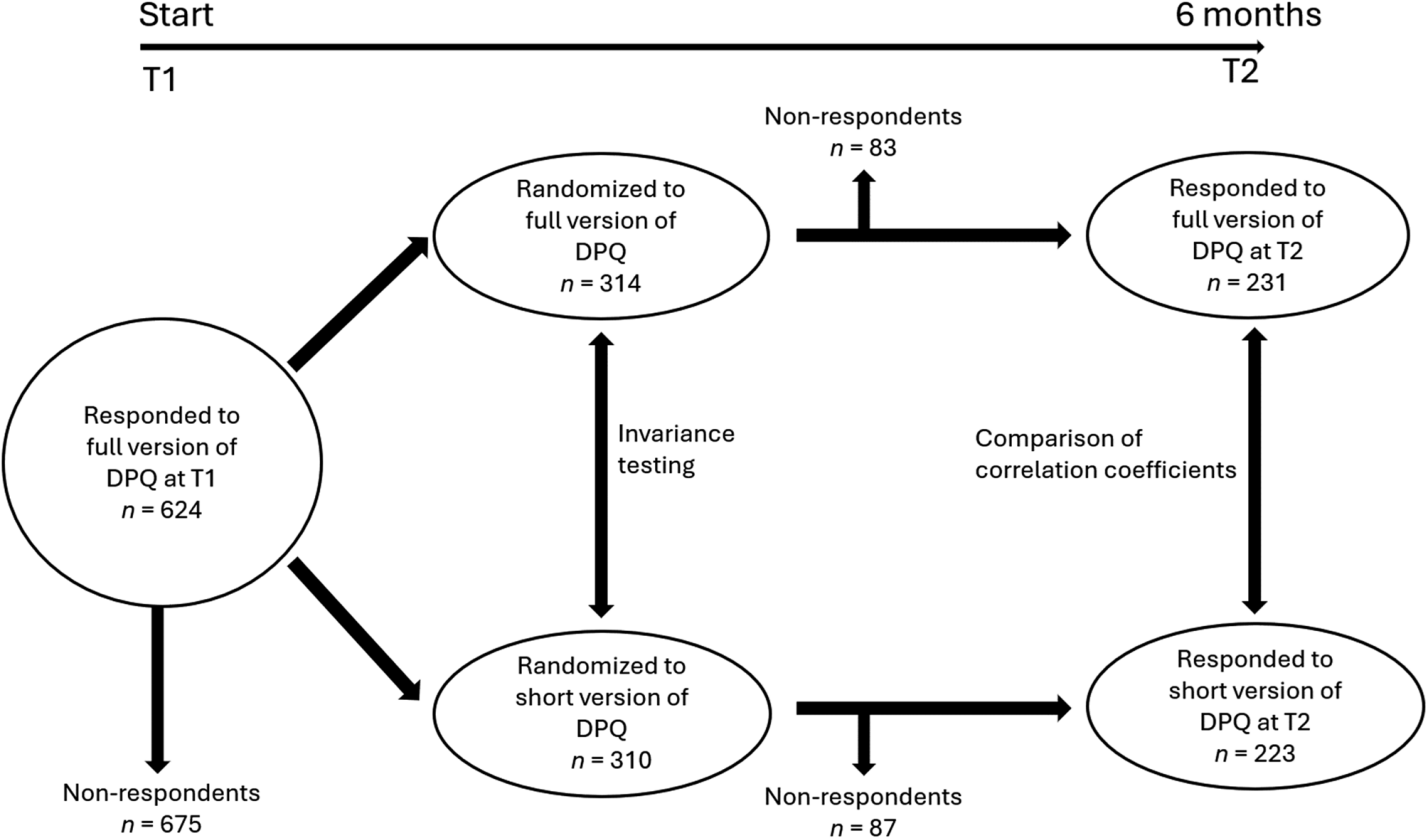
Outline of data collection

In line with recommendations for validation of single items [21], the participants that answered the survey at the initial data collection were randomized on a cohort level, department by department, into one of two groups. At Time 2 (T2), six months after answering the first survey, one group received a survey identical to the first one, while the second group received a single item version of DPQ (except for the dimension *Reactions to the work situation* where all items were included). Once again, the participants had 4 weeks to respond. After the initial request to fill out the survey, six reminders were sent to the participants who had not yet completed the survey. The entire procedure is described in Fig. 1. A total of *N* = 454 participants responded at T2, resulting in a response rate of 73% from T1. See Appendix C.1 and C.2 for tables containing characteristics of the respondents at T2 for the two groups.

### Language validation

The items of DPQ were translated from English to Swedish with a procedure suggested by guidelines for best practice in cross-cultural surveys [22] in which items are translated directly to the intended language (rather than using an indirect procedure such as back-translation) by an expert panel with good understanding of the constructs that the instrument is intended to measure. A group of three native Swedish speakers, with a good understanding of both English and Danish along with solid experience with work environment questionnaires translated the items using both the English and the Danish version of the questionnaire. The group translated the questions independently of each other and then gathered and compared the translations and agreed on the final wording of each item.

Thereafter, the instrument was presented individually to five individuals for a cognitive interview with a think-aloud approach [23]. Two of these individuals were PhD students within clinical psychology, and three were human resources (HR) practitioners within a healthcare organization. The interviewees were instructed to go through the items of the questionnaire while talking about their understanding of the questions. The process only resulted in minor alterations of the questionnaire, such as in which order questions were presented.

### Instrument

The main instrument and focus of the study is the DPQ. As mentioned earlier, the DPQ consists of five dimensions. The dimension concerning negative acts, such as bullying, harassment, and threats of violence, was not included in the validation analyses due to the expected low prevalence of such behaviors in the sample. Low response frequencies can result in restricted variance and skewed distributions, which may reduce the reliability and stability of the analyses necessary for validation [24–26]. It is important to note that for the individuals that are exposed to these types of negative work behaviors, these behaviors are likely the aspects of the work environment that are most harmful to the well-being of the people experiencing them. Hence, these questions are important to include in practical workplace assessments. However, for the purpose of the present validation study, the dimension was considered less suitable for psychometric evaluation.

In Table 2, an overview of the DPQ, including its dimensions, subscales, sample items, number of items within each subscale, and scale reliabilities (McDonald’s omega, ω) for all subscales included in the study, is presented.

**Table 2:** Overview of the DPQ and scale-reliability of each subscale in the present sample

Dimensions and subscales	Sample item^1^	Items *N*	Omega ω
Dimension: demands at work			
Work pace	Do you have to work very fast?	2	N/A
Quantitative demands	How often is it the case that you do not have time to complete all your work tasks?	4	0.83
Emotional demands	Are you placed in emotionally demanding situations at work?	3	0.82
Dimension: work organization and job content			
Role clarity	Do you know exactly what is expected of you at work?	4	0.81
Role conflict	Do you have to do things in your work that you feel should be done differently?	4	0.73
Possibilities for development	Does your work provide you with opportunities for developing your skills?	4	0.79
Predictability	Do you receive timely information about e.g., important decisions, changes and plans for the	4	0.78
Influence at work	Do you have any influence on how you carry out your work tasks?	4	0.82
Possibilities for performing work task	Do your working conditions allow you to carry out your work satisfactorily?	4	0.74
Unnecessary work tasks	Do you have to do work tasks that you think are unnecessary?	4	0.76
Dimension: interpersonal relations: cooperation and leadership			
Changes in the workplace	Did the management inform the employees sufficiently about the changes in the workplace?	4	0.91
Cooperation between colleagues within teams, departments or groups	Do you and your colleagues work well together when problems emerge which require cooperation among you?	4	0.84
Involvement of employees	Does the management encourage you and your colleagues to come up with ideas for improvements?	3	0.90
Justice in the workplace	Does the management at your workplace treat you fairly?	4	0.85
Social Support from management	Can you talk to your immediate supervisor about difficulties you experience at work?	2	N/A
Cooperation with immediate supervisor	Does your immediate supervisor have a clear understanding of the work tasks that you and your co-workers perform?	4	0.87
Trust between colleagues	Do you and your colleagues keep each other informed about things that are important for you to do your job well?	4	0.78
Quality of leadership	Is your immediate supervisor good at motivating the employees?	4	0.89
Dimension: reactions to the work situation			
Experience of meaning at work	Do you think that your work tasks are interesting and inspiring?	4	0.88
Commitment to the workplace	Would you recommend others to apply for a job at your workplace?	4	0.93
Conflict between work and private life	Does your job demand so much of your attention that it has a negative effect on your private life?	3	0.87

^1^See appendix A for a full overview of items including the Swedish translation

### Outcomes

To further validate DPQ, we included outcomes related to health, patient safety, performance and turnover intention in this study. These outcomes are as follows:

### Copenhagen burnout inventory (CBI)

Participants’ self-rated burnout was measured using the 7-item subscale of work-related burnout from the Copenhagen Burnout Inventory (CBI) [27]. Responses to the first three items were given on a 5-point Likert scale from 1 (“to a very low degree”) to 5 (“to a very high degree”) whilst responses to the last four items were given on a 5-point Likert scale from 1 (“never/almost never”) to 5 (“always/almost always”). An example item is “Do you feel worn out at the end of the working day?” Scale-reliability in the present sample was *ω* = 0.90.

### Perceived stress scale 4 (PSS-4)

Participants’ perceived stress was measured through the 4-item Perceived Stress Scale, PSS-4 [28]. Responses were given on a 5-point Likert scale from 1 (“never”) to 5 (“very often”). An example item is “In the last month, how often have you felt that you were unable to control the important things in your life?” Scale-reliability in the present sample was *ω* = 0.76.

### WHO (Five) Well-being index (WHO-5)

Participants’ psychological well-being was assessed through the 5-item WHO (Five) Well-Being Index, WHO-5 [29]. Responses were given on a 6-point Likert scale ranging from 0 (“never”) to 5 (“always”). An example item is “Over the last two weeks: I have felt calm and relaxed”. Scale-reliability in the present sample was *ω* = 0.89.

### Safety attitudes questionnaire (SAQ)

Participants’ subjective assessment of the patient safety climate of their workplace was measured through the 10-item Safety Attitudes Questionnaire (SAQ) [30]. Responses were given on a 5-point Likert scale from 1 (“strongly disagree”) to 5 (“strongly agree”). An example item is “I would feel perfectly safe being treated here as a patient.” Scale-reliability in the present sample was *ω* = 0.83.

### Organizational citizenship behavior (OCB)

Participants’ contextual work performance was assessed through the 5-item altruism subscale within OCB [31]. Responses were given on a 7-point Likert scale from 1 (“do not agree at all”) to 7 (“totally agree”). An example item is “I am always ready to give a helping hand to those around me”. Scale-reliability in the present sample was *ω* = 0.86.

### Work-role performance

Participants’ self-assessed work performance was measured through the 3-item subscale “Individual task proficiency” that is part of the Work-role performance scale [32]. Responses were given on a 5-point Likert scale from 1 (“to a very low degree”) to 5 (“to a very high degree”). An example item is “How often during the last month have you carried out the core parts of your job well?”. Scale-reliability in the present sample was *ω* = 0.82.

### Turnover intention

Participants’ turnover intention was measured through 4 items from the turnover intention scale, TIS-6 [33]. Responses were given on a 5-point Likert scale from 1 (“do not agree at all”) to 5 (“totally agree”). An example item is “I’m considering leaving this organization.” Scale-reliability in the present sample was *ω =* 0.93.

## Results

Analyses were carried out in four phases where the first phase focused on factorial validity and reliability, the second on convergent and discriminant validity. The third phase assessed criterion-based validity, and lastly the fourth phase dealt with instrument reduction and comparison of correlation coefficients. All calculations were conducted with the software program Mplus version 8.10 unless otherwise stated.

### Phase 1 - Reliability and factorial validity

This phase focused on establishing the reliability and factorial validity of the questionnaire. Reliability refers to whether the items within each subscale consistently measure the same underlying construct [34], while factorial validity concerns whether the observed data fit the theoretically expected factor structure – whether items group together into the intended dimensions or subscales as predicted [31].

To test the reliability of the questionnaire, we calculated the Omega (ω) for each subscale within the instrument at T1, where a score > 0.70 suggests good reliability [35]. The omega-value for internal consistency of the subscales in DPQ varied from *ω* = 0.75 to *ω* = 0.93, suggesting good reliability of the instrument. All omega values can be found in Table 1.

We then conducted separate confirmatory factor analysis (CFA) of all individual subscales within the DPQ, see Appendix B. Overall, the results of the CFAs showed that the items loaded on their expected factors with factor loadings that varied between 0.398 − 0.923 for the instrument.

Subsequently, measurement models were estimated to test the validity of DPQ on a dimensional level. A good model fit would be considered when Root Mean Square Error of Approximation (RMSEA) and Standardized Root Mean Square Residual (SRMR) were ≤ 0.08 as well as Comparative Fit Indices (CFI) and Tucker-Lewis Index (TLI) were ≥ 0.90 [26, 36]. To examine the dimensionality of the subscales, we estimated alternative one-factor models and compared them with multifactor models based on the instrument’s division of the subscales into different dimensions. We used the scaled chi-square difference test to compare the models.

Table 3 shows that, for all four dimensions the multi-factor model displayed a significantly better model fit compared to the one-factor model, supporting the factorial validity of DPQ. The dimensions *Demands at work*,* Work organization and job content* and *Interpersonal relations: cooperation and leadership* demonstrated a satisfactory model fit. The dimension *Reactions to the work situation* showed mixed results where the RMSEA-value was somewhat dissatisfactory while the CFI-value was robust.

**Table 3:** Test of model fit of one-factor and multi-factor models for each dimension within DPQ. [χ2 = Chi-square; df=degrees of freedom; RMSEA=root mean square error of approximation; SRMR=standardized root mean square residual; CFI=comparative fit index; TLI=Tucker-Lewis index]

Dimension	Model fit: one-factor model						Model fit: multi factor model				Change in model fit	
	χ2/df	RMSEA	SRMR	CFI	TLI	χ2/df	RMSEA	SRMR	CFI	TLI	Δχ2/Δdf	*P*-value
Demands at work (1 factor vs. 3 factors)	479/27	0.164	0.088	0.814	0.752	67/24	0.053	0.026	0.983	0.974	412/3	< 0.001
Work organization and job content (1 factor vs. 7 factors)	3638/350	0.123	0.109	0.497	0.456	882/329	0.052	0.051	0.915	0.903	2756/21	< 0.001
Interpersonal relations and leadership (1 factor vs. 8 factors)	4977/377	0.140	0.117	0.646	0.619	1303/349	0.066	0.048	0.927	0.915	3674/28	< 0.001
Reactions to the work situation (1 factors vs. 3 factors)	1324/35	0.243	0.123	0.694	0.607	175/32	0.085	0.036	0.966	0.952	1149/3	< 0.001

The measurement models revealed potential overlaps between some of the subscales within DPQ’s dimensions in terms of correlations *r* > 0.85 which has been suggested as a threshold above which values indicate multicollinearity problems [26]. The identified correlations were: *Work pace* and *Quantitative demands* (*r* = 1), *Involvement of employees* and *Justice in the workplace*.

(*r* = 0.95), Social support from management and Cooperation with immediate supervisor (*r* = 0.96), Social support from management and Quality of leadership (*r* = 0.91), Cooperation with immediate supervisor and Quality of leadership (*r* = 1), Cooperation between colleagues within teams, departments or groups and Trust between colleagues (*r* = 1). These results can be found in Appendix D.

### Phase 2: Convergent validity and discriminant validity

Phase 2 aimed to evaluate the convergent and discriminant validity of the questionnaire. Convergent validity concerns whether items within the same subscale measure the same underlying concept and therefore share common variance [37, 38]. Discriminant validity concerns whether constructs are sufficiently differentiated from one another, indicating that the subscales capture separate constructs rather than the same or overlapping concepts [38].

To evaluate convergent and discriminant validity, we calculated the Average Variance Extracted (AVE). Generally, an AVE above 0.50 signifies convergent validity, while discriminant validity is established if the square root of the AVE exceeds the correlations between latent constructs [37]. As presented in Tables 4, 5, and 6, the AVEs for the subscales within the dimensions *demands at work*,* interpersonal relations: cooperation and leadership* and * reactions to the work situation*, were above 0.50, demonstrating convergent validity. In Table 7, which presents the dimension of *work organization and job content*, five of the seven subscales showed an AVE-value below 0.50, which suggests problems regarding the convergent validity of those subscales.

**Table 4:** Latent variable correlations (F) for the study variables within the “demands at work” dimension of DPQ

	1	2	3
Demands at work			
1. Work pace			
2. Quantitative demands	1		
3. Emotional demands	0.67	0.54	
AVE	0.51	0.56	0.63
AVE sqrt	0.72	0.75	0.79

Note. All correlations were *p* < 0.05

**Table 5:** Latent variable correlations (F) for the study variables within the “interpersonal relations: cooperation and leadership” dimension of DPQ

	1	2	3	4	5	6	7	8
Interpersonal relations: cooperation and leadership								
1. Changes in the workplace								
2. Cooperation between colleagues within teams, departments or groups	0.34							
3. Involvement of employees	0.74	0.48						
4. Justice in the workplace	0.81	0.50	0.94					
5. Social support from management	0.43	0.45	0.61	0.64				
6. Cooperation with immediate supervisor	0.49	0.52	0.69	0.72	0.96			
7. Trust between colleagues	0.40	1	0.58	0.66	0.44	0.51		
8. Quality of leadership	0.47	0.50	0.64	0.68	0.91	1	0.50	
AVE	0.70	0.56	0.71	0.57	0.73	0.58	0.51	0.64
AVE sqrt	0.84	0.75	0.85	0.75	0.86	0.76	0.71	0.80

Note. All correlations were *p* < 0.05

**Table 6:** Latent variable correlations (F) for the study variables within the “reactions to the work situation” dimension of DPQ

	1	2	3
Reactions to the work situation			
1. Experience of meaning at work			
2. Commitment to the workplace	0.68		
3. Conflict between work and private life	− .10ns	− 0.30	
AVE	0.63	0.73	0.80
AVE sqrt	0.79	0.86	0.89

Note. All correlations were *p* < 0.05, except those labelled ns

**Table 7:** Latent variable correlations (F) for the study variables within the “work organization and job content” dimension of DPQ

	1	2	3	4	5	6	7
Work organization and job content							
1. Role clarity							
2. Role conflict	− 0.42						
3. Possibilities for development	0.31	− .01ns					
4. Predictability	0.41	− 0.47	0.38				
5. Influence at work	0.34	− 0.24	0.54	0.44			
6. Possibilities for performing work tasks	0.54	− 0.62	0.34	0.54	0.36		
7. Unnecessary work tasks	− 0.41	0.84	− .14ns	− 0.56	− 0.31	− 0.68	
AVE	0.49	0.44	0.54	0.48	0.61	0.47	0.46
AVE sqrt	0.70	0.66	0.74	0.69	0.78	0.68	0.67

Note. All correlations were *p* < 0.05, except those labelled ns

While examining the discriminant validity of the instrument, we observed that the square root of the AVE was higher than the correlations of all subscales, demonstrating discriminant validity, with the exceptions of the following pairs: *Work pace* and *quantitative demands*, *role conflict* and *unnecessary work tasks*, *possibilities for performing work tasks* and *unnecessary work tasks*, *involvement of employees* and *justice in the workplace*, *social support from management* and *cooperation with immediate supervisor*, *social support from management* and *quality of leadership*, *cooperation with immediate supervisor* and *quality of leadership*. These results indicate that discriminant validity is not achieved between multiple subscales, suggesting redundancy within the instrument.

### Phase 3: Criterion-based validity

Phase 3 focused on evaluating the criterion-based validity of the DPQ. Criterion-based validity concerns whether the questionnaire and its subscales are meaningfully associated with external variables or outcomes that they are theoretically expected to relate to, (i.e., high levels of demands and burnout) [20, 39]. It is reflected by statistically significant associations in theoretically expected directions, with stronger associations expected for conceptually related constructs [40]. Demonstrating criterion-based validity provides evidence that the instrument is capable of reflecting relevant real-world outcomes and their connection to the psychosocial work environment and therefore supports the practical usefulness and interpretability of the questionnaire [20]. To establish criterion-based validity, we correlated the DPQ subscales with well-established scales measuring burnout, patient safety, well-being, and performance (OCB), among others. These are presented in Appendix D.

Within the dimension *Demands at work*, we found that the strongest associations were with burnout which aligned with expectations [39]. It is worth noting that patient safety demonstrated a weak association with this dimension.

However, all the subscales within *Work organization and job content* and *Interpersonal relations: cooperation and leadership* showed statistically significant associations with patient safety that varied between *r = − .35 (Role conflict)* and *r* = 0.76 (Trust between colleagues), in line with previous research findings that have found these aspects highly relevant for safer practices in healthcare [41]. Furthermore, all subscales within *Work organization and job content* showed associations with health outcomes such as burnout and wellbeing.

Within the dimension *Interpersonal relations: cooperation and leadership* a notable result is that all subscales except *Changes in the workplace* were statistically significantly associated with the *Organizational citizenship behavior* (OCB) which is consistent with previous findings linking these forms of job resources to performance in healthcare settings [42].

Lastly in the dimension *Reactions to the work situation*, we found that patient safety was associated both with *Experience of meaning at work* and *Commitment to workplace*, supporting the relevance of evaluating these dimensions in a healthcare setting. Among the health variables burnout showed a strong correlation with *Conflicts between work and private life* while well-being was moderately associated with *Experience of meaning at work* and *Commitment to workplace.*

### Phase 4 – Instrument reduction and comparison of correlation coefficients

The procedure of reducing the original instrument into a single-item version was as follows: First, correlations between the different subscales within each dimension were compared and if a high correlation (*r* ≥ .85) was identified one of the subscales was dropped. See appendix B for the correlations between the different subscales. The decision of which subscales to exclude was made by the authors after theoretical discussions anchored in empirical evidence combined with the statistical performance of the subscales. The subscales that were excluded from the single-item version with the rationale behind the decision were as follows:

*Work pace* was dropped and *Quantitative demands* remained in the single-item version. *Work pace* is generally seen as a narrower construct and has typically been included in broader terms such as “work pressure” or “quantitative workload” [43]. This decision was also supported by our data since the AVE-value was lower for *Work pace* (*AVE =* 0.51) compared to *Quantitative demands* (*AVE =* 0.56).

Furthermore, *Role conflict* was excluded in favor of *Unnecessary work tasks*. *Unnecessary work tasks* represent a more specific and actionable job demand, reflecting tasks perceived as non-value-adding [44]. We therefore chose to keep *Unnecessary work tasks* since the construct represents a demand that can be seen as particularly relevant in healthcare settings. For example, due to a substantial proportion of time often being spent on administrative or low-value tasks in combination with an outspoken strategy to minimize this burden for the benefit of freeing up more time to meet patients [45]. The AVE-values of the construct supported our decision since *Role conflict* showed a marginally lower value (*AVE =* 0.44) compared to *Unnecessary work tasks* (*AVE =* 0.46). Note that *Role conflict* did not exceed correlations of *r* ≥ .85 with *Unnecessary work tasks* (*r =* 0.84), but it also correlated above *r* > .60 with both *unnecessary work tasks* and *possibilities for performing work tasks.* Based on this, after deliberation among the authors, *conflict* was also excluded since keeping both constructs for the short instrument could not be justified due to the small amount of additional information it would give.

*Involvement of employees* was omitted and *Justice in the workplace* was selected to be included in the single-item version. Organizational justice has for a long time shown relevance for the psychosocial work environment within occupational health research and has been linked to key outcomes such as worker well-being and occupational stress within healthcare settings [46, 47]. Thus the construct was prioritized as it represents a broader and more theoretically established dimension of the work environment. The AVE-values of the subscales aligned with our decision since *Involvement of employees* showed a marginally lower value (*AVE =* 0.56) compared to *Justice in the workplace* (*AVE =* 0.57).

Cooperation with immediate supervisor and Social support from management was removed from the single-item version in favor of Quality of leadership. Note that Social support from management had the highest AVE-value (AVE = 0.73), compared to Quality of leadership (AVE = 0.64) and Cooperation with immediate supervisor (AVE = 0.58) but as Quality of leadership includes questions concerning both employees motivation and communicating of goals we chose this scale since it focuses more on leadership aspects that are well known to affect the psychosocial work environment [48, 49].

*Trust between colleagues* was the last subscale to be omitted from the single-item version in favor of *Cooperation between colleagues within teams*,* departments or groups. Cooperation between colleagues* was prioritized as it captures a broader and more behaviorally oriented aspect of teamwork, including communication, coordination, and collaborative work processes [50]. Healthcare settings are complex and require a great number of cooperative processes to ensure good care coordination, patient safety, and high-quality care [51]. Although *Trust between colleagues* represents an important component of the psychosocial work environment that enables high-quality teamwork [52, 53] it was considered the narrower of the two constructs and therefore excluded. The AVE-values of the subscales supported our decision since *Trust between colleagues* demonstrated a lower value (*AVE =* 0.51) compared to *Cooperation between colleagues within teams*,* departments or groups.* (*AVE =* 0.56).

The selected items within each subscale were then compared statistically using corrected item - total correlation (CI-TC) calculations in which a higher value is preferable as this indicates that the item explains more variance in the answers for the subscale. Calculations were made using the software program IBM SPSS Statistics version 29.0.1.0(171). The CI-TC value in combination with a theoretical viability of how the item represented the latent construct was chosen for the single-item version. All the chosen items demonstrated a higher CI-TC value except for the subscale *Justice in the workplace* where the item *“Does the management at your workplace treat you fairly?”* (*CI-TC =* 0.71) was chosen over the item **“***Does the management at your workplace respect you?” (CI-TC = 0.75)* and within the subscale *Quantitative demands* where the item “*Do you get behind with your work?”* (*CI-TC =* 0.67) was chosen over the item *“How often do you have deadlines that are hard to meet?”* (*CI-TC =* 0.68). See appendix E for all the items chosen for the single-item version.

After constructing the single-item version, all employees who responded at T1 were randomized at the cohort level, department by department, to either complete the full instrument or the single-item version at the 6-month follow-up. We proceeded with invariance testing of the two groups to determine whether full scalar invariance could be achieved on a dimensional level between the two groups. This was done to ensure that there were no differences between the two groups that would make it difficult to compare their results with each other. Based on Chen’s recommendations [54] when the sample size is *N* > 300 the change in Comparative Fit Index (CFI) should be less than 0.01 between metric, configural, and scalar which was achieved in our sample. Therefore, we can conclude that there were no significant differences between the two groups regarding their response patterns at T1. The CFI values of the invariance testing can be seen in Table 8.

**Table 8:** Invariance testing of both groups at T1

Dimension	Comparative Fit Index (CFI)		
	Configural	Metric	Scalar
Demands at work	0.992	0.993	0.994
Work organization and job content	0.909	0.912	0.913
Interpersonal relations: cooperation and leadership	0.921	0.922	0.921
Reactions to the work situation	0.966	0.970	0.971

We then compared the correlation coefficients over time for each construct for both groups using MedCalc version 22.023 to examine whether they were similar, and hence the single-item version could be a sufficient alternative to the full instrument. When comparing the correlations between T1 and T2 for both groups, significant differences for all subscales were found between the group that had the full version of the DPQ and the group that received the single-item version. Hence, the single-item version is not a suitable option for use compared to the original version. The correlation differences, Z-statistics, significance levels (p), and 95% confidence intervals can be found in Appendix F.

## Discussion

This study aimed to translate the DPQ and evaluate its reliability and validity when used to assess the psychosocial work environment for healthcare personnel in Sweden. The English and Danish versions of DPQ were translated into Swedish using an expert panel and psychometric analyses established that the Swedish version overall demonstrates good reliability comparable with the validation of the original Danish version of the instrument [13].

When examining the factorial validity, the DPQ showed a good fit within all dimensions that the instrument is structured around. This confirms the findings from the original validation of the instrument and that the theoretical division of the content of the instrument seems to be solid.

However, the results of the validation demonstrated problems with high intercorrelations (above *r >* 0.85) between subscales and in some cases even a perfect correlation (e.g. *Work pace* and *Quantitative demands*,* r =* 1). These findings were also complemented by lack of discriminant validity between the subscales. This suggests that some subscales which theoretically represent different phenomena in the psychosocial work environment overlap instead of capturing unique variance, creating statistical redundancy in the instrument. A model may demonstrate good overall fit because of how the broader organization of items and dimensions corresponds to the observed data. However, a good or acceptable model fit does not necessarily imply that all latent constructs within the model are empirically distinct from one another. In the present study, the high correlations suggest that some theoretically separate subscales may capture very similar experiences for respondents. In this sense, the DPQ may still be useful as a multidimensional framework for describing the psychosocial work environment, while some individual subscales should be interpreted with caution because they appear to provide partly overlapping information.

However, this is a known problem for several constructs typically used in psychosocial work environment surveys. For example, the close connection between the constructs *Work pace* and *Quantitative demands* has been recognized as overlapping and also sensitive to the type of work, (e.g., blue-collar work being more sensitive to work pace compared to white-collar work [55]. Additionally, in line with our results, previous research has shown that separating leadership into narrower constructs often encounters this multicollinearity problem [56]. Therefore, a way forward for the development of the DPQ could be to see if these findings are present in other study settings and if these results are consistent, the next step would be to exclude subscales that are redundant from the instrument.

Another concern is the dimension *Work organization and job content* where the majority of the subscales demonstrated an AVE-value under 0.50 indicating that there could be problems with the convergent validity within this dimension. This indicates the need for further examination of the dimension in future research, as well as caution in analyses based on the use of these scales.

Since one of the aims of this study was to validate DPQ in a healthcare setting with healthcare professionals we were particularly interested in how the instrument would associate with patient safety since safety culture has been shown to be a reliable predictor for both the psychological health and engagement for healthcare professionals as well as positive patient outcomes [41, 57]. Interestingly, patient safety did not show a strong association with the dimension *Demands at work* but was found to have stronger connections to subscales within the resource dimensions, *work organization and job content* and *Interpersonal relations: cooperation and leadership* as well as the outcome dimension *Reactions to the work situation.* This implies that, to achieve a good safety culture in healthcare, interventions aimed at reducing workload may not be the primary focus. Rather, interventions that enhance job resources, such as collaboration between colleagues or employee involvement in decision-making, could be more effective. Another explanation for this finding could be that healthcare personnel over the last decades have been facing increasingly higher demands. Thus, making a strainful work environment “a new normal” in which they sacrifice their own wellbeing to uphold high quality care for patients.

Another aim of the study was to investigate if DPQ could be shortened to achieve greater time efficiency for organizations that want to use it. This was done by excluding subscales that had a very high (*r* > .85) correlation with other parts of the instrument and letting the remaining subscales be represented by single-item questions. When comparing correlation coefficients for T1 and T2 of the full version and the single-item version we found statistical differences between the versions, suggesting that single-item questions for the dimensions in DPQ are not a suitable approach for reducing the instrument. Problems with validity and the use of subjective single items to measure psychological constructs is something that has been discussed previously [21]. Aspects such as cooperation in teams, social interaction between employees and management and the organization and structure of work tasks are complex phenomena where a combination of questions is necessary to get a fair representation of these phenomena using a questionnaire. However, our results also show that some subscales have a very high intercorrelation with each other.

### Limitations and future research

As with all research, there are several limitations to the present study. Among these, it is important to note that we are unaware of the distribution of age, sex, profession, and tenure of the 52% of the invited healthcare workers who did not respond to the initial survey. Therefore, we are unable to perform a non-response analysis and detect potential bias among respondents in the sample. Consequently, it cannot be ruled out that the respondents differed systematically from non-respondents, which may influence the representativeness of the sample and should be considered when interpreting these validation results.

A further limitation is that the construction of the single-item version partly involved expert judgement. Although the reduction procedure was guided by empirical indicators, including intercorrelations, AVE-values, and corrected item-total correlations, alternative reduction strategies based on other statistical approaches or theoretical assumptions might have resulted in a somewhat different selection of retained constructs and items.

In addition, the data collection was performed at the end of 2021 and in late spring of 2022 when the healthcare sector was heavily affected by the COVID-19 pandemic and its aftermath. It is possible that the strain of dealing with this crisis affected the response patterns of the entire sample - or of specific professions in a way that would not have been the case if the data had been collected at another time. As mentioned earlier we would like to see further validation of the DPQ in other healthcare settings. The study population is restricted to one healthcare organization in one region in Sweden, and therefore the results may not be generalizable to a wider context. Thus, we suggest that the study should be replicated in a wider Swedish healthcare context to reveal whether results are influenced by the pandemic’s aftermath and specific characteristics of the population.

## Conclusion

This study translated and validated the Danish Psychosocial Work Environment Questionnaire (DPQ) for use in a Swedish healthcare context. The Swedish version of the DPQ demonstrated good reliability, comparable to the original Danish version.

The factorial validity supported the multifactor structure of the DPQ, indicating a strong goodness-of-fit within all dimensions. However, some subscales showed high intercorrelations, suggesting potential overlaps in what they measure, which could lead to unnecessary redundancy if the aim is to assess the work environment, raising questions about the validity of the instrument. However, all the subscales and their items can also be very useful as means for risk assessments, discussions, and a base for potential interventions aimed at improving the work environment in workplaces, which is the primary reason for the development of DPQ.

Interestingly, patient safety (SAQ) was more strongly associated with job resources (e.g., work organization, interpersonal relations) than with job demands. This suggests that interventions aimed at improving patient safety should focus on enhancing job resources rather than merely reducing workload. However, high job demands were strongly linked to burnout, highlighting the need for balanced interventions that address both demands and resources.

The study also explored the feasibility of a shortened version of the DPQ using single-item measures. The results indicated that the proposed single-item version performed significantly worse than the full instrument and therefore cannot currently be recommended as a substitute for the original version. Rather than supporting implementation of a single-item version, the findings highlight the methodological difficulties involved in reducing complex psychosocial work environment constructs to single-item measures.

Our results indicate that the Swedish version of the DPQ is a feasible tool for assessing the psychosocial work environment in healthcare settings. Researchers are recommended to consider the potential redundancy within the instrument as well as convergent validity problems within the dimension *Work organization and job content*. Future research should aim to explore the reliability and validity of the Swedish version in a broader healthcare context, for example with multiple healthcare organizations.

## Supplementary Information

Below is the link to the electronic supplementary material.

Supplementary Material 1

## Data Availability

Data will be made availiable upon reasonable request from the corresponding author.

## Abbreviations

AVE: Average Variance Extracted
CBI: Copenhagen Burnout Inventory
CFA: Confirmatory Factor Analysis
CFI: Comparative Fit Index
CI: Confidence Interval
CI-TC: Corrected Item-Total Correlation
df: Degrees of freedom
DPQ: Danish Psychosocial Work Environment Questionnaire
HR: Human Resources
M: Mean
N: Number of participants
N/A: Not applicable
ns: Non-significant
OCB: Organizational Citizenship Behavior
p: Probability value
PSS-4: Perceived Stress Scale 4
r: Correlation coefficient
RMSEA: Root Mean Square Error of Approximation
SAQ: Safety Attitudes Questionnaire
SD: Standard deviation
SPSS: Statistical Package for the Social Sciences
SRMR: Standardized Root Mean Square Residual
T1: Time 1
T2: Time 2
TIS-6: Turnover Intention Scale 6
TLI: Tucker-Lewis Index
WHO-5: World Health Organization-Five Well-Being Index
χ²: Chi-square
Δχ²: Change in chi-square
Δdf: Change in degrees of freedom
ω: McDonald’s omega
