# Supplementary material for: Validation of the Danish Psychosocial Questionnaire (DPQ) in a Swedish healthcare context
Source: BMC Health Serv Res. 2026 Jul 13;26:977. doi: 10.1186/s12913-026-15083-z (PMC13371244; doi:10.1186/s12913-026-15083-z)
Supplement: Supplementary file 1 — Supplementary Material 1 [file 12913_2026_15083_MOESM1_ESM.docx]

**APPENDIX A**This appendix contains is the items from the Danish Psychosocial and work environment Questionnaire (DPQ) used in this study. In the first and second columns we find the English and Danish items and in the third column we find the Swedish version which was translated in this study.

Domain: Demands at work

1.1 Work Pace

| 1 Do you have to work very fast? | Er det nødvendigt at arbejde meget hurtigt? | Är du tvungen att arbeta väldigt snabbt? |
| --- | --- | --- |
| 2 Is the pace of work so fast that it affects the quality of your work? | Er arbejdstempoet så højt, at det påvirker kvaliteten af dit arbejde? | Är arbetstempot så högt att det påverkar kvaliteten i ditt arbete? |
| Response options: “Always”; “Often”; “Sometimes”; “Rarely”; “Never/almost never” |  | Alltid, Ofta, Ibland, Sällan, Aldrig/Nästan aldrig |

1.2 Quantitative demands

| 1 How often is it the case that you do not have time to complete all your work tasks? | Hvor ofte sker det, at du ikke når alle dine arbejdsopgaver? | Har du tillräckligt med tid för att utföra dina arbetsuppgifter? |
| --- | --- | --- |
| 2 How often do you receive unscheduled work tasks that place you under time pressure? | Hvor ofte får du uventede arbejdsopgaver, der sætter dig under tidspres? | Hur ofta får du ta emot oväntade arbetsuppgifter som sätter dig under tidspress? |
| 3 How often do you have deadlines that are hard to meet? | Hvor ofte har du tidsfrister, der er svære at overholde? | Hur ofta har du ’deadlines’ som är svåra att klara av? |
| 4 Do you get behind with your work? | Kommer du bagud med dit arbejde? | Kommer du efter med ditt arbete? |
| Response options: “Always”; “Often”; “Sometimes”; “Rarely”; “Never/almost never” |  | Alltid, Ofta, Ibland, Sällan, Aldrig/Nästan aldrig |

1.3 Emotional demands

| 1 Are you placed in emotionally demanding situations at work? | Bringer dit arbejde dig i følelsesmæssigt krævende situationer? | Hamnar du genom ditt arbete i känslomässigt påfrestande situationer? |
| --- | --- | --- |
| 2 As a result of your work, do you come into contact with people who oppose you or are aggressive towards you? | Har du i dit arbejde kontakt til personer, der er modvillige eller aggressive over for dig? | Har du i ditt arbete kontakt med människor som är motvilliga eller aggressiva mot dig? |
| 3 Do you have to deal with relationships at work that are emotionally challenging? | Har du i forbindelse med dit arbejde relationer, der er følelsesmæssigt vanskelige at håndtere? | Behöver du hantera relationer som är känslomässigt krävande i ditt arbete? |
| Response options: “Always”; “Often”; “Sometimes”; “Rarely”; “Never/almost never” |  | Alltid, Ofta, Ibland, Sällan, Aldrig/Nästan aldrig |

Domain: Work organization and job content

2.1 Influence at work

| 1. Do you have any influence on how you carry out your work tasks? | Har du indflydelse på, hvordan du løser dine arbejdsopgaver? | Kan du påverka hur du utför dina arbetsuppgifter? |
| --- | --- | --- |
| 2 Do you have sufficient authority to deal with the responsibilities you have in your work? | Har du tilstrækkelige beføjelser i forhold til det ansvar, du har i dit arbejde? | Har du tillräckligt inflytande över dina ansvarsområden i ditt arbete? |
| 3.Is it possible for you to make important decisions about your work? | Har du mulighed for at træffe væsentlige beslutninger om dit arbejde? | Kan du fatta viktiga beslut om ditt arbete? |
| 4.Do you have any influence on the order in which you carry out your work tasks? | Har du indflydelse på i hvilken rækkefølge, du løser dine arbejdsopgaver? | Kan du påverka i vilken ordning du utför dina arbetsuppgifter? |
| Response options: “To a very large extent”; “To a large extent”; “Somewhat”; “To a small extent”; “To a very small extent” |  | I mycket hög grad, I hög grad, Delvis, I låg grad, I mycket låg grad |

2.2 Possibilities for development

| 1 Does your work provide you with opportunities for developing your skills? | Giver dit arbejde dig muligheder for at udvikle dine kompetencer? | Erbjuder ditt arbete möjligheter att utveckla dina färdigheter? |
| --- | --- | --- |
| 2 Do your work tasks vary a lot? | Har du meget varierede arbejdsopgaver? | Är ditt arbete varierat? |
| 3 Do you have possibilities to learn something new through your work? | Har du muligheder for at lære noget nyt gennem dit arbejde? | Har du möjlighet att lära dig något nytt genom ditt arbete? |
| 4 Do you have good opportunities for further training and education? | Har du gode muligheder for efter- og videreuddannelse? | Har du goda möjligheter till vidareutbildning? |
| Response options: “To a very large extent”; “To a large extent”; “Somewhat”; “To a small extent”; “To a very small extent” |  | I mycket hög grad, I hög grad, Delvis, I låg grad, I mycket låg grad |

2.3 Role Clarity

| 1 Are there clear goals for your work tasks? | Er der klare mål for dit eget arbejde? | Finns det klara mål för ditt arbete? |
| --- | --- | --- |
| 2 Do you know exactly what is expected of you at work? | Ved du nøjagtigt, hvad der forventes af dig i dit arbejde? | Vet du precis vad som förväntas av dig i ditt arbete? |
| 3 Do you know when you have carried out your job well? | Ved du, hvornår du har udført dit arbejde godt? | Vet du när du utfört ditt arbete väl? |
| 4 Do you know exactly what your responsibilities are? | Ved du helt klart, hvad der er dine ansvarsområder? | Vet du exakt vilka som är dina ansvarsområden? |
| Response options: “To a very large extent”; “To a large extent”; “Somewhat”; “To a small extent”; “To a very small extent” |  | I mycket hög grad, I hög grad, Delvis, I låg grad, I mycket låg grad |

2.4 Role conflicts

| 1 Do you have to do things in your work that you feel should be done differently? | Skal du gøre ting i dit arbejde, som du føler, burde gøres anderledes? | Måste du ibland göra något som egentligen borde ha gjorts annorlunda? |
| --- | --- | --- |
| 2 Are there any conflicting demands in your work? | Bliver der stillet modstridende krav til dig i dit arbejde? | Ställs det krav som strider mot varandra i ditt arbete? |
| 3 Does your job involve tasks that conflict with your personal values? | Indeholder dit arbejde opgaver, der er i modstrid med dine personlige værdier? | Innefattar ditt arbete arbetsuppgifter som är i konflikt med dina personliga värderingar? |
| 4 Do you sometimes have to end a task even though you do not feel you have completed it? | Må du nogen gange afslutte en opgave, før du føler, du er færdig med den? | Måste du ibland avsluta en uppgift trots att du inte är klar med den? |
| Response options: “To a very large extent”; “To a large extent”; “Somewhat”; “To a small extent”; “To a very small extent” |  | I mycket hög grad, I hög grad, Delvis, I låg grad, I mycket låg grad |

2.5 Possibilities for performing work tasks

| 1 Do your working conditions allow you to carry out your work satisfactorily? | Giver forholdene på din arbejdsplads mulighed for, at du kan udføre arbejdet tilfredsstillende? | Tillåter dina arbetsförhållanden dig att utföra ditt arbete på ett tillfredsställande sätt? |
| --- | --- | --- |
| 2 Do you have the tools you need (e.g. technical assistive devices, tools, machinery, IT solutions, etc.) for you to do your job satisfactorily? | Har du de redskaber, du har behov for (fx hjælpemidler, værktøj, maskiner, IT-løsninger mv.), så du kan udføre arbejdet tilfredsstillende? | Har du de hjälpmedel (t ex teknisk utrustning, verktyg, maskiner, IT-lösningar m.m.) som behövs för att utföra ditt arbete på ett tillfredsställande sätt? |
| 3 Are there enough employees at work for you to do your job satisfactorily? | Er der nok medarbejdere på arbejde til, at du kan udføre dit arbejde tilfredsstillende? | Är det tillräckligt med personal på din arbetsplats för att du ska kunna utföra ditt arbete på ett tillfredsställande sätt? |
| 4 Can you perform your work tasks to a level of quality that you are satisfied with? | Kan du udføre dit arbejde i en kvalitet, du er tilfreds med? | Kan du utföra ditt arbete med en kvalitet som du är nöjd med? |
| Response options: “To a very large extent”; “To a large extent”; “Somewhat”; “To a small extent”; “To a very small extent” |  | I mycket hög grad, I hög grad, Delvis, I låg grad, I mycket låg grad |

2.6 Unnecessary work tasks

| 1.Do you spend time on work tasks that you have difficulty seeing the purpose with? | Bruger du tid på arbejdsopgaver, som du har svært ved at se formålet med? | Lägger du tid på arbetsuppgifter som du har svårt att se syftet med? |
| --- | --- | --- |
| 2. Are you placed in situations at work that are unnecessarily difficult to deal with? | Bliver du på dit arbejde sat i situationer, der er unødvendigt vanskelige? | Hamnar du i situationer på ditt arbete som är onödigt svåra att hantera? |
| 3. Is your work made more difficult than necessary due to poor work procedures? | Bliver dit arbejde besværliggjort af uhensigtsmæssige arbejdsgange? | Blir ditt arbete svårare än nödvändigt på grund av bristfälliga rutiner? |
| 4. Do you have to do work tasks that you think are unnecessary? | Skal du udføre arbejdsopgaver, som du synes er unødvendige? | Måste du utföra arbetsuppgifter du anser är onödiga? |
| Response options: “To a very large extent”; “To a large extent”; “Somewhat”; “To a small extent”; “To a very small extent” |  | I mycket hög grad, I hög grad, Delvis, I låg grad, I mycket låg grad |

2.7 Predictability

| 1 Do you receive timely information about e.g. important decisions, changes and plans for the future at your place of work? | Får du på din arbejdsplads informationer om fx vigtige beslutninger, ændringer og fremtidsplaner i god tid? | Får du information i god tid på din arbetsplats t.ex. när det gäller viktiga beslut, förändringar och framtidsplaner? |
| --- | --- | --- |
| 2 Are you informed well in advance if changes are made to your work tasks? | Bliver du informeret i god tid, hvis der sker ændringer i dine arbejdsopgaver? | Får du information i god tid om dina arbetsuppgifter förändras? |
| 3 Are you informed well in advance of changes to whom you will be working with? | Bliver du informeret i god tid, hvis der sker ændringer i, hvem du skal arbejde sammen med? | Får du information i god tid om vem du arbetar med förändras? |
| 4 Are you informed well in advance if there are changes to your working hours? | Bliver du informeret i god tid, hvis der sker ændringer i dine arbejdstider? | Får du information i god tid om din arbetstid förändras? |
| Response options for Q1: “To a very large extent”;“To a large extent”;“Somewhat”;“To a small extent”;“To a very small extent”. Response options for Q2-4: “Always”; “Often”; “Sometimes”; “Rarely”; “Never/almost never” |  | Item 1: I mycket hög grad, I hög grad, Delvis, I låg grad, I mycket låg grad Item 2-4: Alltid, Ofta, Ibland, Sällan, Aldrig/Nästan aldrig |

Domain: Interpersonal relations: cooperation and leadership

3.1 Cooperation between colleagues within teams, departments, or groups

| 1 Do you and your colleagues help each other if someone has too much to do? | Hjælper I kolleger, der har for meget at lave, der hvor du arbejder? | Hjälper du och dina kollegor varandra om någon har för mycket att göra? |
| --- | --- | --- |
| 2 Is there a sense of community and cohesion between you and your colleagues? | Er der en følelse af sammenhold og samhørighed blandt dig og dine kolleger? | Finns det en känsla av gemenskap och sammanhållning mellan dig och dina kollegor? |
| 3 Do you and your colleagues agree on what is most important in your work tasks? | Er du og dine kolleger enige om, hvad der er det vigtigste i jeres arbejdsopgaver? | Är du och dina kollegor överens om vad som är viktigast bland era arbetsuppgifter? |
| 4 Do you and your colleagues work well together when problems emerge which require cooperation among you? | Er du og dine kolleger gode til at samarbejde, når der opstår problemer, der kræver fælles løsninger? | Arbetar du och dina kollegor bra ihop när problem uppstår som kräver att ni samarbetar? |
| Response options: “To a very large extent”; “To a large extent”; “Somewhat”; “To a small extent”; “To a very small extent” |  | I mycket hög grad, I hög grad, Delvis, I låg grad, I mycket låg grad |

3.2 Trust between colleagues

| 1 Do you and your colleagues keep each other informed about things that are important for you to do your job well? | Holder du og dine kolleger hinanden underrettede om ting, der er vigtige for, at I kan udføre arbejdet godt? | Håller du och dina kollegor varandra informerade om saker som är viktiga för att ni ska kunna göra ett bra jobb? |
| --- | --- | --- |
| 2 Do you trust the ability of your colleagues to do their job well? | Har du tillid til dine kollegers evne til at gøre arbejdet godt? | Litar du på dina kollegors förmåga till att göra ett bra jobb? |
| 3 Can you express your views and feelings to your closest colleagues? | Kan du give udtryk for dine meninger og følelser over for dine nærmeste kolleger? | Kan du uttrycka dina åsikter och känslor till dina närmaste kollegor? |
| 4 In general, do you and your colleagues trust one another? | Stoler du og dine kolleger i almindelighed på hinanden? | Litar de anställda i allmänhet på varandra? |
| Response options: “To a very large extent”; “To a large extent”; “Somewhat”; “To a small extent”; “To a very small extent” |  | I mycket hög grad, I hög grad, Delvis, I låg grad, I mycket låg grad |

3.3 Cooperation with immediate supervisor

| 1 Is the relationship between your immediate supervisor and you and your co-workers characterized by mutual respect and recognition? | Er forholdet mellem din nærmeste leder og medarbejderne præget af gensidig respekt og anerkendelse? | Kännetecknas relationen mellan din närmaste chef och dig och dina kollegor av ömsesidig respekt och erkännande? |
| --- | --- | --- |
| 2 Does your immediate supervisor have a clear understanding of the work tasks that you and your co-workers perform? | Har din nærmeste leder stor forståelse for det arbejde, medarbejderne udfører? | Har din närmaste chef en tydlig förståelse för de arbetsuppgifter som du och dina kollegor utför? |
| 3 Does your immediate supervisor take the needs and views of you and your co-workers into consideration when making decisions? | Tager din nærmeste leder hensyn til medarbejdernes behov og synspunkter, når han eller hun træffer beslutninger? | Tar din närmaste chef hänsyn till dig och dina kollegors behov och synpunkter när beslut fattas? |
| 4 Does your immediate supervisor contribute to solving everyday problems? | Bidrager din nærmeste leder til at løse konkrete problemer i hverdagen? | Bidrar din närmaste chef till att lösa konkreta problem i vardagen? |
| Response options: “To a very large extent”; “To a large extent”; “Somewhat”; “To a small extent”; “To a very small extent” |  | I mycket hög grad, I hög grad, Delvis, I låg grad, I mycket låg grad |

3.4 Quality of leadership

| 1 Does your immediate supervisor give high priority to the wellbeing of employees in the workplace? | Prioriterer din nærmeste leder trivslen på arbejdspladsen højt? | I vilken utsträckning anser du att din närmaste chef – prioriterar trivseln på arbetsplatsen högt? |
| --- | --- | --- |
| 2 Is your immediate supervisor good at communicating clear goals for the work of you and your colleagues? | Er din nærmeste leder god til at kommunikere klare mål for arbejdet? | I vilken utsträckning anser du att din närmaste chef – kommunicerar tydliga mål i arbetet. |
| 3 Is your immediate supervisor good at resolving conflicts? | Er din nærmeste leder god til at løse konflikter? | I vilken utsträckning anser du att din närmaste chef – är bra på att hantera konflikter? |
| 4 s your immediate supervisor good at motivating the employees? | Er din nærmeste leder god til at motivere medarbejderne? | I vilken utsträckning anser du att din närmaste chef - är bra på att motivera medarbetare? |
| Response options: “To a very large extent”; “To a large extent”; “Somewhat”; “To a small extent”; “To a very small extent” |  | I mycket hög grad, I hög grad, Delvis, I låg grad, I mycket låg grad |

3.5 Social support from management

| 2 Can you talk with your immediate supervisor about difficulties you experience at work? | Kan du tale med din nærmeste leder om det, hvis du oplever problemer i arbejdet? | Kan du prata med din närmaste chef om problem du upplever I arbetet? |
| --- | --- | --- |
| 4 Can you get advice and guidance from your immediate supervisor if you need it? | Kan du få råd og vejledning af din nærmeste leder, hvis du får brug for det? | Kan du få råd och vägledning av din närmaste chef om du behöver det? |
| Response options: “To a very large extent”; “To a large extent”; “Somewhat”; “To a small extent”; “To a very small extent” |  | I mycket hög grad, I hög grad, Delvis, I låg grad, I mycket låg grad |

3.6 Justice in the workplace

| 1 Are conflicts resolved in a fair way? | Bliver konflikter løst på en retfærdig måde? | Löses konflikter på ett rättvist sätt? |
| --- | --- | --- |
| 2 Can one get a clear reason when important decisions are made in your workplace? | Kan man få en klar begrundelse, når der træffes væsentlige beslutninger på din arbejdsplads? | Ges en tydlig motivering när det fattas viktiga beslut på din arbetsplats? |
| 3 Does the management at your workplace respect you? | Bliver du respekteret af ledelsen på din arbejdsplads? | Respekteras du av ledningen på din arbetsplats? |
| 4 Does the management at your workplace treat you fairly? | Bliver du behandlet retfærdigt af ledelsen på din arbejdsplads? | Behandlas du rättvist på din arbetsplats? |
| Response options: “To a very large extent”; “To a large extent”; “Somewhat”; “To asmall extent”; “To a very small extent” |  | I mycket hög grad, I hög grad, Delvis, I låg grad, I mycket låg grad |

3.7 Involvement of employees

| 1 Does the management encourage you and your colleagues to come up with ideas for improvements? | Opmuntrer ledelsen dig og dine kolleger til at komme med ideer til forbedringer? | Uppmuntrar ledningen dig och dina kollegor att komma med förslag på förbättringar? |
| --- | --- | --- |
| 2 Do employees and managers work well together to improve work procedures? | Er medarbejdere og ledere gode til at samarbejde om at forbedre arbejdsgangene? | Arbetar anställda och chefer på ett bra sätt för att tillsammans förbättra arbetsrutiner? |
| 3 Are suggestions for improvements treated seriously by the management in the workplace? | Bliver forslag til forbedringer behandlet seriøst af ledelsen på arbejdspladsen? | Tas förslag till förbättringar på allvar av ledningen på arbetsplatsen? |
| Response options: “To a very large extent”; “To a large extent”; “Somewhat”; “To a small extent”; “To a very small extent” |  | I mycket hög grad, I hög grad, Delvis, I låg grad, I mycket låg grad |

3.8 Recognition

| 1 Are your efforts recognized and appreciated at your place of work? | Bliver din arbejdsindsats anerkendt og påskønnet på din arbejdsplads? | Blir de anställda uppskattade för en bra arbetsinsats? |
| --- | --- | --- |
| Response options: “To a very large extent”; “To a large extent”; “Somewhat”; “To a small extent”; “To a very small extent” |  | I mycket hög grad, I hög grad, Delvis, I låg grad, I mycket låg grad |

3.9 Changes in the workplace

Filter question:

| 1 Have any major changes been implemented at your workplace during the last two years (e.g. a restructuring of the workplace or layoffs)? | om de har oplevet større forandringer på arbejdspladsen inden for det seneste år (fx en omorganisering af arbejdspladsen eller en fyringsrunde). | Har större förändringar genomförts på din arbetsplats under de senaste två åren? (t. ex. omstrukturering eller uppsägningar) |
| --- | --- | --- |
| Response options: “Yes, several times”; “Yes, one time”; “No” |  | Ja flera gånger, Ja, en gång, Nej |
| If Yes, the following questions should be asked: |  | Om “Ja” ska följade frågor ställas: |
| 2 Did the management inform the employees sufficiently about the changes in the workplace? | Har ledelsen informeret medarbejderne tilstrækkeligt om forandringerne på arbejdspladsen? | Informerade ledningen anställda tillräckligt om förändringarna på arbetsplatsen? |
| 3 Have the employees been sufficiently involved in relation to the changes? | Er medarbejderne blevet inddraget tilstrækkeligt i forbindelse med forandringerne? | Involverades de anställda tillräckligt i samband med förändringarna? |
| 4 Are you generally satisfied with the way the management dealt with the changes | Er du generelt tilfreds med den måde, ledelsen har håndteret forandringerne på? | Är du överlag nöjd med sättet ledningen hanterade förändringarna? |
| Do you understand the management's reasons for implementing the changes? | Har du forståelse for ledelsens begrundelser for at gennemføre forandringerne? | Förstår du ledningens skäl för att genomföra förändringarna? |
| Response options: “To a very large extent”; “To a large extent”; “Somewhat”; “To a small extent”; “To a very small extent” |  | I mycket hög grad, I hög grad, Delvis, I låg grad, I mycket låg grad |

Domain: Conflicts in the workplace

4.1 Threats

| 1 Have you been exposed to work-related threats during the last 12 months? | Har du inden for de sidste 12 måneder været udsat for trusler i forbindelse med dit arbejde? | Har du under de senaste 12 månaderna blivit utsatt för hot om våld på din arbetsplats? |
| --- | --- | --- |
| ('Threats' denotes verbal or written threats or threatening behaviour) Response options: “Yes, daily or almost daily”; “Yes, weekly”; “Yes, monthly”; “Yes, occasionally”; “No” |  | Ja, dagligen  Ja, varje vecka  Ja, varje månad  Ja, några gånger  Nej |
| 2.If yes, who were you threatened by? |  | Om ja, från vem?  (Det går att markera med flera kryss) |
| Response options: “Overall management/Business owner”; “Immediate supervisor”; “Colleagues”; “Subordinates”; “Customers,clients, patients, pupils, relatives (with ‘relatives’ we think of relatives to pupils, clients or patients)”; “The threat has/threats have been put forward anonymously” |  | Kollegor En överordnad  Underställda  Klienter/ kunder/ patienter |

4.2 Violence

| 1 Have you been exposed to work-related physical violence during the last 12 months? | Har du inden for de sidste 12 måneder været udsat for fysisk vold i forbindelse med dit arbejde? | Har du under de senaste 12 månaderna blivit utsatt för fysiskt våld på din arbetsplats? |
| --- | --- | --- |
| Response options: “Yes, daily or almost daily”; “Yes, weekly”; “Yes, monthly”; “Yes, occasionally”; “No” |  | Ja, dagligen  Ja, varje vecka  Ja, varje månad  Ja, några gånger  Nej |
| 2.If yes, who was violent towards you? |  | Om ja, från vem?  (Det går att markera med flera kryss) |
| Response options: “Overall management/Business owner”; “Immediate supervisor”; “Colleagues”; “Subordinates”; “Customers,clients, patients, pupils, relatives (with ‘relatives’ we think of relatives to pupils, clients or patients)” |  | Kollegor En överordnad  Underställda  Klienter/ kunder/ patienter |

4.3 Bullying

| 1 Have you been exposed to bullying in your current job during the last 12 months? (Bullying takes place when a person repeatedly and over an extended period of time is exposed to unpleasant or degrading treatment. For bullying to take place the person who is bullied must find it difficult to defend him-or herself.) | Har du været udsat for mobning på din nuværende arbejdsplads inden for de sidste 12 måneder? | Har du under de senaste 12 månaderna blivit utsatt för mobbing på din arbetsplats? |
| --- | --- | --- |
| Response options: “Yes, daily or almost daily”; “Yes, weekly”; “Yes, monthly”; “Yes, occasionally”; “No” |  | Ja, dagligen, Ja, varje vecka, Ja, varje månad, Ja, några gånger, Nej |
| 2 f yes, who were you bullied by? |  | Om ja, från vem? (Det går att markera med flera kryss) |
| Response options: “Overall management/Business owner”; “Immediate supervisor”; “Colleagues”; “Subordinates”; “Customers, clients, patients, pupils, relatives (with ‘relatives’ we think of relatives to pupils, clients or patients)” |  | Kollegor, En överordnad, Underställda, Klienter/kunder/patienter |

4.4 Sexual harassment

| 1 Have you been exposed to sexual harassment in your workplace during the last 12 months? | Har du inden for de sidste 12 måneder været udsat for seksuel chikane på din arbejdsplads? | Har du under de senaste 12 månaderna blivit utsatt för icke önskvärd sexuell uppmärksamhet på din arbetsplats? |
| --- | --- | --- |
| Response options: “Yes, daily or almost daily”; “Yes, weekly”; “Yes, monthly”; “Yes, occasionally”; “No” |  | Ja, dagligen, Ja, varje vecka, Ja, varje månad, Ja, några gånger, Nej |
| 2 If yes,who were you sexually harassed by? |  | Om ja, från vem? (Det går att markera med flera kryss) |
| Response options: “Overall management/Business owner”; “Immediate supervisor”; “Colleagues”; “Subordinates”; “Customers, clients, patients, pupils, relatives (with ‘relatives’ we think of relatives to pupils, clients or patients)” |  | Kollegor, En överordnad, Underställda, Klienter/kunder/patienter |

4.5 Discrimination

| 1 Have you within the last 12 months experienced discrimination or been treated poorly due to e.g. your sex, age, ethnicity, religion, health or sexual orientation? | Har du inden for de sidste 12 måneder oplevet at blive diskrimineret eller dårligt behandlet på din arbejdsplads på grund af dit køn, din alder, din etnicitet, din religion, dit helbred eller din seksuelle orientering? | Har du under de senaste 12 månaderna upplevt diskriminering eller blivit illa behandlad på grund av till exempel ditt kön, ålder, etnicitet, religion, funktionsnedsättning, sexuell läggning eller könsöverskridande identitet/uttryck? |
| --- | --- | --- |
| Response options: “Yes, daily or almost daily”; “Yes, weekly”; “Yes, monthly”; “Yes, occasionally”; “No” |  | Ja, dagligen  Ja, varje vecka  Ja, varje månad  Ja, några gånger  Nej |
| 2 If yes, who discriminated or treated you poorly? |  | Om ja, från vem? (Det går att markera med flera kryss) |
| If yes, who discriminated or treated you poorly? Response options: “Overall management/Business owner”; “Immediate supervisor”; “Colleagues”; “Subordinates”; “Customers, clients, patients, pupils, relatives (with ‘relatives’ we think of relatives to pupils, clients or patients)” |  | Kollegor  En överordnad  Underställda  Klienter/kunder/patienter |

4.6 Harassment

| 1 Have you within the last 12 months experienced work-related harassment by customers, clients, patients, pupils or relatives? (Harassment occurs when a person is exposed to offensive acts, threats or persecution from persons that one is in contact with through one's job, e.g. customers, clients, patients, pupils or their relatives, but not colleagues, superiors or subordinates) | Har du inden for de sidste 12 måneder oplevet at blive chikaneret af kunder, klienter, patienter, elever eller pårørende i forbindelse med dit arbejde? | Har du under de senaste 12 månaderna upplevt arbetsrelaterade trakasserier från kunder, klienter, elever, patienter eller deras anhöriga? (Arbetsrelaterade trakasserier uppstår när en person utsätts för kränkande handlingar, hot eller förföljelse från personer som man är i kontakt med genom sitt jobb, till exempel kunder, klienter, elever, patienter eller deras anhöriga, men inte kollegor, överordnade eller underordnade) |
| --- | --- | --- |
| Response options: “Yes, daily or almost daily”; “Yes, weekly”; “Yes, monthly”; “Yes, occasionally”; “No” |  | Ja, dagligen, Ja, varje vecka, Ja, varje månad, Ja, några gånger, Nej |
| 2 If yes, how/where did the harassment occur? |  | Om ja, hur/var inträffade trakasserierna? |
| Response options: “At my workplace”; “Outside of my workplace, e.g. at home or in town”; “Via social media”; “By telephone, SMS, email or letter”; “Other” |  | På min arbetsplats, utanför min arbetsplats (t.ex. hemma eller på stan), Via sociala medier, Via telefon, sms, epost eller brev, annat |

Domain: Reactions to the work situation

5.1 Experience of meaning at work

| 1 Do you feel motivated and engaged in your work? | Føler du dig motiveret og engageret i dit arbejde? | Känner du dig motiverad och engagerad i ditt arbete? |
| --- | --- | --- |
| 2 Are your work tasks meaningful? | Er dine arbejdsopgaver meningsfulde? | Är ditt arbete meningsfullt? |
| 3 Do you think that your work tasks are interesting and inspiring? | Synes du, at dine arbejdsopgaver er interessante og inspirerende? | Tycker du att dina arbetsuppgifter är intressanta och inspirerande? |
| 4 Does your work give you self-confidence and job satisfaction? | Giver dit arbejde dig selvtillid og arbejdsglæde? | Ger ditt arbete dig självförtroende och arbetsglädje? |
| Response options: “To avery large extent”; “To a large extent”; “Somewhat”; “To a small extent”; “To a very small extent” |  | I mycket hög grad, I hög grad, Delvis, I låg grad, I mycket låg grad |

5.2 Commitment to the workplace

| 1 Would you recommend others to apply for a job at your workplace? | Vil du anbefale andre at søge en stilling på din arbejdsplads? | Skulle du rekommendera andra att söka anställning på din arbetsplats? |
| --- | --- | --- |
| 2 Do you tell your friends that your workplace is a good place to work? | Fortæller du dine venner, at din arbejdsplads er et godt sted at arbejde? | Berättar du för dina vänner att din arbetsplats är ett bra ställe att jobba på? |
| 3 Are you proud of working at your workplace? | Er du stolt over at arbejde på din arbejdsplads? | Är du stolt över att jobba på din arbetsplats? |
| 4 Does your workplace inspire you to do your best? | Inspirerer din arbejdsplads dig til at yde dit bedste? | Min arbetsplats inspirerar mig verkligen att göra mitt bästa |
| Response options: “To a very large extent”; “To a large extent”; “Somewhat”; “To a small extent”; “To a very small extent” |  | I mycket hög grad, I hög grad, Delvis, I låg grad, I mycket låg grad |

5.5 Self-reported stress

| 1 How often have you felt stressed within that last two weeks? | Hvor ofte har du følt dig stresset inden for de sidste to uger? | Hur ofta har du känt dig stressad under de senaste två veckorna? |
| --- | --- | --- |
| Response options: “All the time”; “Often”; “Sometimes”; “Rarely”; “Never” |  | Alltid, Ofta, Ibland, Sällan, Aldrig |
| If “All the time”,“Often”,“Sometimes”, or “Rarely”,the following questions should be asked: |  | Om “Alltid, “Ofta”, “Ibland” eller “sällan” ska följande frågor ställas: |
| 2 What was the most significant source of your stress? | hvad den vigtigste kilde var til, at de følte sig stressede | Vad var den främsta orsaken till att du kände dig stressad? |
| Response options: “Work”; “Private life”; “Both work and private life” |  | Arbetet, Privatlivet, Både arbetet och privatlivet |

5.8 Conflict between work-life and private life

| 1 Does your job demand so much of your energy that it has a negative effect on your private life? | Tager dit arbejde så meget af din energi, at det går ud over privatlivet? | Känner du att ditt arbete tar så mycket av din energi att det påverkar privatlivet negativt? |  |
| --- | --- | --- | --- |
| 2 Does your job demand so much of your time that it has a negative effect on your private life? | Tager dit arbejde så meget af din tid, at det går ud over privatlivet? | Känner du att ditt arbete tar så mycket av din tid att det påverkar privatlivet negativt? |  |
| Response options: “To a very large extent”; “To a large extent”; “Somewhat”; “To a small extent”; “To a very small extent” |  | I mycket hög grad, I hög grad, Delvis, I låg grad, I mycket låg grad |  |

5.6 Job satisfaction

| 1 Overall, how satisfied are you with your job? | Hvor tilfreds er du med dit job som helhed, alt taget i betragtning? | Hur tillfredsställd är du med ditt arbete som helhet, allt inräknat? |
| --- | --- | --- |
| Response options: Ascale from 0 to 10, where 0 denotes the lowest possible level of job satisfaction and 10 denotes the highest possible level of job satisfaction. |  | Svarsalternativ: 0-10 där 0 innebär lägsta möjliga nivå av tillfredsställelse och 10 innebär högsta möjliga nivå av tillfredsställelse. |

**APPENDIX B**

*Factor loadings for items in DPQ*

| Factor loadings (L) | L item 1 | L item 2 | L item 3 | L item 4 |
| --- | --- | --- | --- | --- |
| *Quantatative Demands* |  |  |  |  |
| Work Pace | 0.630 | 0.833 | **-** | **-** |
| Quantatative Demands | 0.736 | 0.668 | 0.773 | 0.760 |
| Emotional Demands | 0.803 | 0.672 | 0.837 | **-** |
| *Work organization and job content* |  |  |  |  |
| Role Clarity | 0.730 | 0.923 | 0.739 | 0.440 |
| Role Conflict | 0.651 | 0.806 | 0.545 | 0.518 |
| Possibilities for Development | 0.843 | 0.538 | 0.802 | 0.616 |
| Predictability | 0.657 | 0.830 | 0.697 | 0.569 |
| Influence at work | 0.795 | 0.824 | 0.645 | 0.663 |
| Possibilities for Performing Work task | 0.755 | 0.398 | 0.685 | 0.821 |
| Unnecessary Work Tasks | 0.623 | 0.651 | 0.617 | 0.402 |
| *Interpersonal relations: cooperation and leadership* |  |  |  |  |
| Changes in the work place | 0.856 | 0.884 | 0.915 | 0.704 |
| Cooperation between colleagues within teams, departments or groups | 0.701 | 0.802 | 0.698 | 0.798 |
| Involvement of employees | 0.798 | 0.774 | 0.766 | 0.824 |
| Justice in the workplace | 0.648 | 0.861 | 0.768 | 0.778 |
| Social Support from Management | 0.796 | 0.912 | **-** | **-** |
| Cooperation with immediate supervisor | 0.798 | 0.774 | 0.766 | 0.824 |
| Trust between colleagues | 0.581 | 0.654 | 0.705 | 0.800 |
| Quality of leadership | 0.841 | 0.788 | 0.787 | 0.877 |
| *Reactions to the work situation* |  |  |  |  |
| Experience of Meaning at work | 0.820 | 0.684 | 0.839 | 0.843 |
| Commitment to the workplace | 0.885 | 0.858 | 0.914 | 0.806 |
| Conflict between work and private life | N/A | N/A | **-** | **-** |

**Appendix C.1**

*Characteristics of respondents of the full version of DPQ at T2.*

| Characteristics *N*(%) |  | Surgery center | Laboratory | Habilitation center | Primary care | Total |
| --- | --- | --- | --- | --- | --- | --- |
| Sex |  |  |  |  |  |  |
| Female |  | 54 (83,1%) | 58 (72,5%) | 41 (91,1%) | 34 (82,9%) | 187 (81,0%) |
| Male |  | 11 (16,1%) | 22 (27,5%) | 4 (8,9%) | 7 (17,1%) | 44 (19,0%) |
| Other/missing |  | - | - | - | - | - |
|  |  |  |  |  |  |  |
| Age mean (sd) |  | 43,8 (12,4) | 45,4 (12,0) | 49,7 (9,7) | 48,7 (11,0) | 46,4 (11,7) |
|  |  |  |  |  |  |  |
| Degree |  |  |  |  |  |  |
| Primary School |  | 1 (1,5%) | - | 1 (2,2%) | 1 (2,4%) | 3 (1,3%) |
| High school |  | 18 (27,7%) | 9 (11,3%) | 3 (6,7%) | 11 (26,8%) | 41 (17,7%) |
| University |  | 44 (67,7%) | 62 (77,5%) | 39 (86,7%) | 29 (70,7%) | 174 (75,3%) |
| PhD |  | 2 (3,1%) | 9 (11,3%) | 1 (2,2%) | - | 12 (5,2%) |
| Missing |  | - | - | 1 (2,2%) | - | 1 (0,4%) |
|  |  |  |  |  |  |  |
| Occupation |  |  |  |  |  |  |
| Nurse |  | 25 (38,5%) | 4 (5,0%) | - | 8 (19,9%) | 37 (16,0%) |
| Assistant nurse |  | 16 (24,6%) | 2 (2,5%) | - | 9 (22,0%) | 27 (11,7%) |
| Physician |  | 8 (12,3%) | 11 (13,8%) | - | 5 (12,2%) | 24 (10,4%) |
| Psychologist/Counselor | | 1 (0,5%) | - | - | 14 (31,1%) | 4 (9,8%) |
| Manager |  | 3 (4,6%) | 6 (7,5%) | 4 (8,9%) | 4 (9,8%) | 17 (7,4%) |
| Medical secretary |  | 5 (7,7%) | 2 (2,5%) | - | 2 (4,9%) | 9 (3,9%) |
| Care administrator |  | 4 (6,2%) | 3 (3,8%) | 6 (13,3%) | 4 (9,8%) | 17 (7,4%) |
| Fysiotherapist |  | - | - | 3 (6,7%) | 3 (7,3%) | 6 (2,6%) |
| Biomedicinal analyst/Lab techician | | - | - | 52 (65%) | - | - |
| Occupational therapist | | 1 (0,5%) | 1 (1,5%) | - | 7 (15,6%) | 1 (2,4%) |
| Other |  | 3 (4,6%) | - | 11 (24,4%) | 1 (2,4%) | 15 (6,5%) |
|  |  |  |  |  |  |  |
| Years in organization | |  |  |  |  |  |
| <3 years |  | 8 (12,3%) | 11 (13,8%) | 6 (9,2%) | 5 (12,2%) | 30 (13,0%) |
| 3-13 years |  | 34 (52,3%) | 34 (42,5%) | 18 (27,7%) | 18 (43,9%) | 104 (45,0%) |
| >13 years |  | 23 (35,4%) | 34 (42,5%) | 21 (32,3%) | 18 (43,9%) | 96 (41,6%) |
| Missing |  | - | 1 (1,3%) | - | - | 1 (0,4%) |
|  |  | 65 | 80 | 45 | 41 | 231 |

**Appendix C.2**

*Characteristics of respondents of the short single-item version of DPQ at T2.*

| Characteristics N(%) |  | Surgery center | Laboratory | Habilitation center | Primary care | Total |
| --- | --- | --- | --- | --- | --- | --- |
| Sex |  |  |  |  |  |  |
| Female |  | 51 (76,1%) | 64 (81%) | 33 (89,2%) | 37 (92,5%) | 185 (83%) |
| Male |  | 15 (22,4%) | 14 (17,7%) | 4 (10,8%) | 3 (7,5%) | 36 (16,1%) |
| Other/missing |  | 1 (1,5%) | 1 (1,3%) | - | - | 2 (0,9%) |
|  |  |  |  |  |  |  |
| Age mean (sd) |  | 44,7 (12,7) | 46,0 (11,7) | 48,9 (9,2) | 46,2 (11,3) | 46,1 (11,6) |
|  |  |  |  |  |  |  |
| Degree |  |  |  |  |  |  |
| Primary School |  | - | - | - | - | - |
| High school |  | 11 (16,4%) | 7 (8,9%) | 1 (2,7%) | 10 (25,0%) | 29 (13,0%) |
| University |  | 51 (76,1%) | 52 (65,8%) | 36 (97,3%) | 30 (75,0%) | 169 (75,8%) |
| PhD |  | 4 (6,0%) | 20 (25,3%) | - | - | 24 (10,8%) |
| Missing |  | 1 (1,5%) | - | - | - | 1 (0,4%) |
|  |  |  |  |  |  |  |
| Occupation |  |  |  |  |  |  |
| Nurse |  | 28 (41,8%) | 6 (7,6%) | 1 (2,7%) | 12 (30%) | 47 (21,1%) |
| Assistant nurse |  | 7 (10,4%) | - | - | 6 (15%) | 13 (5,8%) |
| Physician |  | 12 (17,9%) | 9 (11,4%) | - | 1 (2,5%) | 22 (9,9%) |
| Psychologist/Counselor | | 1 (0,5%) | 1 (1,5%) | - | 16 (43,2%) | 3 (7,5%) |
| Manager |  | 5 (7,5%) | 6 (7,6%) | - | 6 (15,0%) | 17 (7,6%) |
| Medical secretary |  | 8 (11,9%) | 2 (2,5%) | - | 4 (10,0%) | 14 (6,3%) |
| Care administrator |  | 2 (3,0%) | 2 (2,5%) | 2 (5,4%) | 6 (15,0%) | 12 (5,4%) |
| Fysiotherapist |  | 2 (3,0%) | - | 4 (10,8%) | 1 (2,5%) | 7 (3,1%) |
| Biomedicinal analyst/Lab techician | | - | - | 54 (68,4%) | - | - |
| Occupational therapist | | 1 (0,5%) | - | - | 7 (18,9%) | 1 (2,5%) |
| Other |  | 2 (3,0%) | - | 7 (18,9%) | - | 9 (4,0%) |
|  |  |  |  |  |  |  |
| Years in organization | |  |  |  |  |  |
| <3 years |  | 11 (16,4%) | 11 (13,9%) | 8 (21,6%) | 11 (27,5%) | 41 (18,4%) |
| 3-13 years |  | 26 (38,8%) | 38 (48,1%) | 15 (40,5%) | 13 (32,5%) | 92 (41,3%) |
| >13 years |  | 30 (44,8%) | 30 (38,0%) | 14 (37,8%) | 15 (37,5%) | 89 (39,9%) |
| Missing |  | - | - | - | 1 (2,5%) | 1 (0,4%) |
|  |  | 67 | 79 | 37 | 40 | 223 |

**APPENDIX D***Intercorrelations of study variables*

| Subscale | 1 | 2 | 3 | 4 | 5 | 6 | 7 | 8 | 9 | 10 | 11 |
| --- | --- | --- | --- | --- | --- | --- | --- | --- | --- | --- | --- |
| 1 Work Pace | - |  |  |  |  |  |  |  |  |  |  |
| 2 Quantatative Demands | 1.00** | - |  |  |  |  |  |  |  |  |  |
| 3 Emotional Demands | 0.67** | 0.54** | - |  |  |  |  |  |  |  |  |
| 4 Role Clarity | -0.23* | -0.29** | -0.27** | - |  |  |  |  |  |  |  |
| 5 Role Conflict | 0.74** | 0.68** | 0.75** | -0.42** | - |  |  |  |  |  |  |
| 6 Possibilities for Development | 0.02 | 0.08 | 0.01 | 0.31** | -0.01 | - |  |  |  |  |  |
| 7 Predictability | -0.39** | -0.41** | -0.23** | 0.41** | -0.46** | 0.38** | - |  |  |  |  |
| 8 Influence at work | -0.11 | -0.06 | 0.02 | 0.34** | -0.27* | 0.54** | 0.44** | - |  |  |  |
| 9 Possibilities for Performing Work task | -0.80** | -0.78** | -0.56** | 0.54** | -0.62** | 0.34** | 0.54** | 0.36** | - |  |  |
| 10 Unnecessary Work Tasks | 0.74** | 0.61** | 0.72** | -0.41** | 0.84** | -0.14 | -0.56** | -0.31** | -0.68** | - |  |
| 11 Changes in the work place | -0.27** | -0.24** | -0.20* | 0.25* | -0.38** | 0.39** | 0.66** | 0.45** | 0.42** | -0.38** | - |
| 12 Cooperation between colleagues within teams. departments or groups | -0.08 | -0.14 | -0.07 | 0.50* | -0.19 | 0.46** | 0.39** | 0.34** | 0.37** | -0.18* | 0.34** |
| 13 Involvement of employees | -0.30** | -0.21* | -0.25** | 0.49** | -0.43** | 0.49** | 0.61** | 0.50** | 0.49** | -0.47** | 0.74** |
| 14 Justice in the workplace | -0.32** | -0.24** | -0.28** | 0.51** | -0.39** | 0.49** | 0.70** | 0.55** | 0.52** | -0.46** | 0.81** |
| 15 Social Support from Management | -0.16 | -0.10 | -0.13 | 0.34** | -0.17 | 0.38** | 0.48** | 0.28** | 0.26** | -0.18 | 0.43** |
| 16 Cooperation with immediate supervisor | -0.25* | -0.24* | -0.21* | 0.48** | -0.24* | 0.38** | 0.55** | 0.31** | 0.42** | -0.28** | 0.49** |
| 17 Trust between colleagues | -0.14 | -0.14 | -0.06 | 0.47** | -0.12 | 0.47** | 0.48** | 0.44** | 0.37** | -0.24* | 0.40** |
| 18 Quality of leadership | -0.19* | -0.20* | -0.11 | 0.44** | -0.18* | 0.37** | 0.47** | 0.29** | 0.37** | -0.20* | 0.47** |
| 19 Experience of Meaning at work | -0.03 | -0.05 | -0.12 | 0.56** | -0.26* | 0.59** | 0.38** | 0.54** | 0.44** | -0.24* | 0.30** |
| 20 Commitment to the workplace | -0.28** | -0.20* | -0.16* | 0.49** | -0.34** | 0.52** | 0.48** | 0.51** | 0.53** | -0.35* | 0.51** |
| 21 Conflict between work and private life | 0.73** | 0.71** | 0.49** | -0.19* | 0.50** | -0.01 | -0.34** | -0.12 | -0.50** | 0.51** | -0.21** |
| 22 Patient Safety | -0.22* | -0.16 | -0.21* | 0.63** | -0.35** | 0.59** | 0.46** | 0.55** | 0.46** | -0.41** | 0.49** |
| 23 Percieved stress scale | 0.48** | 0.50** | 0.33** | -0.13 | 0.32** | -0.10 | -0.27** | -0.09 | -0.46** | 0.34** | -0.12 |
| 24 WHO-5 | -0.42** | -0.46** | -0.32** | 0.38** | -0.33** | 0.33** | 0.38** | 0.35** | 0.55** | -0.32** | 0.29** |
| 25 Turnover Intention | 0.45** | 0.34** | 0.33** | -0.26** | 0.42** | -0.25** | -0.36** | -0.17* | -0.41** | 0.35** | -0.33** |
| 26 Copenhagen Burnout Inventory | 0.77** | 0.70** | 0.57** | -0.32** | 0.58** | -0.17* | -0.48** | -0.25** | -0.66** | 0.64** | -0.36** |
| 27 Work role Performance | -0.11 | -0.23* | -0.18* | 0.46** | -0.26* | 0.11 | 0.14 | 0.08 | 0.48** | -0.17 | 0.11 |
| 28 Organizational citizen behaviour scale | 0.02 | -0.04 | 0.09 | 0.48** | -0.10 | 0.22* | 0.21* | 0.22* | 0.29** | -0.07 | 0.18 |
| Subscale | 12 | 13 | 14 | 15 | 16 | 17 | 18 | 19 | 20 | 21 | 22 |
| 12 Cooperation between colleagues within teams. departments or groups | - |  |  |  |  |  |  |  |  |  |  |
| 13 Involvement of employees | 0.48** | - |  |  |  |  |  |  |  |  |  |
| 14 Justice in the workplace | 0.50** | 0.95** | - |  |  |  |  |  |  |  |  |
| 15 Social Support from Management | 0.45** | 0.61** | 0.64** | - |  |  |  |  |  |  |  |
| 16 Cooperation with immediate supervisor | 0.52** | 0.69** | 0.72** | 0.96** | - |  |  |  |  |  |  |
| 17 Trust between colleagues | 1.00** | 0.58** | 0.66** | 0.44** | 0.51** | - |  |  |  |  |  |
| 18 Quality of leadership | 0.50** | 0.64** | 0.68** | 0.91** | 1.00** | 0.50** | - |  |  |  |  |
| 19 Experience of Meaning at work | 0.53** | 0.49** | 0.54** | 0.31** | 0.40** | 0.59** | 0.35** | - |  |  |  |
| 20 Commitment to the workplace | 0.58** | 0.68** | 0.71** | 0.58** | 0.62** | 0.62** | 0.63** | 0.68** | - |  |  |
| 21 Conflict between work and private life | -0.11 | -0.20* | -0.23** | -0.10 | -0.21* | -0.11 | -0.20* | -0.10 | -0.30** | - |  |
| 22 Patient Safety | 0.69** | 0.65** | 0.70** | 0.49** | 0.54** | 0.76** | 0.50** | 0.59** | 0.69** | -0.12 | - |
| 23 Percieved stress scale | -0.26** | -0.07 | -0.20* | -0.06 | -0.13 | -0.22* | -0.11 | -0.12 | -0.25** | 0.57** | -0.15 |
| 24 WHO-5 | 0.44** | 0.35** | 0.44** | 0.18* | 0.29** | 0.43** | 0.30** | 0.44** | 0.47** | -0.65** | 0.41** |
| 25 Turnover Intention | -0.26** | -0.07 | -0.20* | -0.06 | -0.13 | -0.22* | -0.11 | -0.12 | -0.25** | 0.57** | -0.15 |
| 26 Copenhagen Burnout Inventory | 0.44** | 0.35** | 0.44** | 0.18* | 0.29** | 0.43** | 0.30** | 0.44** | 0.47** | -0.65** | 0.41** |
| 27 Work role Performance | -0.29** | -0.40** | -0.42** | -0.34** | -0.39** | -0.30** | -0.38** | -0.35** | -0.62** | 0.38** | -0.28** |
| 28 Organizational citizen behaviour scale | -0.26** | -0.39** | -0.44** | -0.23** | -0.34** | -0.27** | -0.31** | -0.30** | -0.46** | 0.89** | -0.31** |

| Subscale | 23 | 24 | 25 | 26 | 27 | 28 |
| --- | --- | --- | --- | --- | --- | --- |
| 23 Percieved stress scale | - |  |  |  |  |  |
| 24 WHO-5 | -0.67** | - |  |  |  |  |
| 25 Turnover Intention | 0.33** | -0.38** | - |  |  |  |
| 26 Copenhagen Burnout Inventory | 0.62** | -0.74** | 0.55** | - |  |  |
| 27 Work role Performance | -0.15 | 0.23** | -0.04 | -0.16 | - |  |
| 28 Organizational citizen behaviour scale | -0.23** | 0.29** | -0.06 | -0.07 | 0.46** | - |
| *Note. Subscales within the dimensions of DPQ: Demands at work (1-3), Work organization and job content (4-10), Interpersonal relations: cooperation and leadership (11-18), Reactions to the work situation (19-21).  Variables 22-28 are outcome variables not included in DPQ* | | | | | | |
|  | | | | | | |
|  | | | | | | |

**APPENDIX E**

In this appendix the results of the inter item consistency calculations using corrected item - total correlation (CI-TC) is presented.

The items chosen for each subscale of the single item version of DPQ is also shown by **bold text**.

| Subscale | Items | CI-TC |
| --- | --- | --- |
| Changes in the workplace | Did the management inform the employees sufficiently about the changes in the workplace? | 0,79 |
|  | Have the employees been sufficiently involved in relation to the changes? | 0,83 |
|  | **Are you generally satisfied with the way the management dealt with the changes** | 0,87 |
|  | Do you understand the management's reasons for implementing the changes? | 0,67 |
|  |  |  |
| Cooperation between colleagues within teams, departments or groups | Do you and your colleagues help each other if someone has too much to do? | 0,63 |
|  | **Is there a sense of community and cohesion between you and your colleagues?** | 0,72 |
|  | Do you and your colleagues agree on what is most important in your work tasks? | 0,62 |
|  | Do you and your colleagues work well together when problems emerge which require cooperation among you? | 0,71 |
|  |  |  |
| Emotional demands | Are you placed in emotionally demanding situations at work? | 0,69 |
|  | As a result of your work, do you come into contact with people who oppose you or are aggressive towards you? | 0,61 |
|  | **Do you have to deal with relationships at work that are emotionally challenging?** | 0,71 |
|  |  |  |
| Justice in the workplace | Are conflicts resolved in a fair way? | 0,61 |
|  | Does the management at your workplace respect you? | 0,75 |
|  | Can one get a clear reason when important decisions are made in your workplace? | 0,70 |
|  | **Does the management at your workplace treat you fairly?** | 0,71 |
|  |  |  |
| Possibilities for development | **Does your work provide you with opportunities for developing your skills?** | 0,70 |
|  | Do your work tasks vary a lot? | 0,47 |
|  | Do you have possibilities to learn something new through your work? | 0,68 |
|  | Do you have good opportunities for further training and education? | 0,54 |
|  |  |  |

| Subscale | Items | CI-TC |  |
| --- | --- | --- | --- |
| Predictability | Do you receive timely information about e.g. important decisions, changes and plans for the future at your place of work? | 0,55 |  |
|  | **Are you informed well in advance if changes are made to your work tasks?** | 0,68 |  |
|  | Are you informed well in advance of changes to whom you will be working with? | 0,61 |  |
|  | Are you informed well in advance if there are changes to your working hours? | 0,53 |  |
|  |  |  |  |
| Possibilities for performing work tasks | Do your working conditions allow you to carry out your work satisfactorily? | 0,63 |  |
|  | Do you have the tools you need (e.g. technical assistive devices, tools, machinery, IT solutions, etc.) for you to do your job satisfactorily? | 0,36 |  |
|  | Are there enough employees at work for you to do your job satisfactorily? | 0,55 |  |
|  | **Can you perform your work tasks to a level of quality that you are satisfied with?** | 0,65 |  |
|  |  |  |  |
| Quantatative demands | How often is it the case that you do not have time to complete all your work tasks? | 0,65 |  |
|  | How often do you receive unscheduled work tasks that place you under time pressure? | 0,60 |  |
|  | How often do you have deadlines that are hard to meet? | 0,68 |  |
|  | **Do you get behind with your work?** | 0,67 |  |
|  |  |  |  |
| Quality of leadership | Does your immediate supervisor give high priority to the wellbeing of employees in the workplace? | 0,79 |  |
|  | Is your immediate supervisor good at communicating clear goals for the work of you and your colleagues? | 0,74 |  |
|  | Is your immediate supervisor good at resolving conflicts? | 0,73 |  |
|  | **Is your immediate supervisor good at motivating the employees?** | 0,81 |  |
|  |  |  |  |
| Role conflicts | Do you have to do things in your work that you feel should be done differently | 0,52 |  |
|  | **Are there any conflicting demands in your work?** | 0,61 |  |
|  | Does your job involve tasks that conflict with your personal values? | 0,46 |  |
|  | Do you sometimes have to end a task even though you do not feel you have completed it? | 0,46 |  |
|  |  |  |  |
| Subscale | | Items | CI-TC |
| Role clarity | Are there clear goals for your work tasks? | 0,65 |  |
|  | **Do you know exactly what is expected of you at work?** | 0,76 |  |
|  | Do you know when you have carried out your job well? | 0,64 |  |
|  | Do you know exactly what your responsibilities are? | 0,43 |  |

| Unnecessary work tasks | Do you spend time on work tasks that you have difficulty seeing the purpose with? | 0,50 |
| --- | --- | --- |
|  | Are you placed in situations at work that are unnecessarily difficult to deal with? | 0,58 |
|  | Is your work made more difficult than necessary due to poor work procedures? | 0,52 |
|  | **Do you have to do work tasks that you think are unnecessary?** | 0,63 |

**APPENDIX F**

*Test of difference of correlation coefficients (*Δ*r) between T1 and T2 for full version and short version of DPQ.*

| Subscale | Pearson’s *r* full version | Pearson’s *r* short version | Δ*r* | z-statistic | *p*-value |
| --- | --- | --- | --- | --- | --- |
|  | *(n =* 231) | *(n =* 223) |  |  |  |
| Emotional Demands | 0,97 | 0,80 | 0,17 | 9,96 | 0,00 |
| Quantatative demands | 0,94 | 0,67 | 0,27 | 9,74 | 0,00 |
| Role Clarity | 0,86 | 0,63 | 0,23 | 5,70 | 0,00 |
| Role Conflict | 0,76 | 0,63 | 0,13 | 2,75 | 0,01 |
| Possibilites for development | 0,84 | 0,65 | 0,19 | 4,81 | 0,00 |
| Predictability | 0,89 | 0,49 | 0,40 | 9,46 | 0,00 |
| Possibilites for performing work tasks | 0,88 | 0,59 | 0,30 | 7,61 | 0,00 |
| Unnecessary work tasks | 0,83 | 0,36 | 0,47 | 8,47 | 0,00 |
| Changes in the workplace | 0,69 | 0,36 | 0,33 | 4,95 | 0,00 |
| Cooperation with Colleagues | 0,78 | 0,59 | 0,18 | 3,75 | 0,00 |
| Justice in the workplace | 0,77 | 0,42 | 0,35 | 6,02 | 0,00 |
| Quality of leadership | 0,67 | 0,55 | 0,12 | 1,99 | 0,05 |
